# Inhibition of Surface-Originated Degradations in Lithium-Rich Layered Cathode via a Pre-Constructed Carbon/Fluorine-Rich Artificial CEI Layer

**DOI:** 10.1007/s40820-026-02276-8

**Published:** 2026-07-10

**Authors:** He Zhao, Jiaqi Sun, Razium Ali Soomro, Ning Sun, Song Hong, Bin Xu

**Affiliations:** 1 State Key Laboratory of Organic-Inorganic Composites, Beijing Key Laboratory of Electrochemical Process and Technology for Materials, Beijing University of Chemical Technology, Beijing, 100029 People’s Republic of China; 2 Institute of Advanced Energy Storage Materials and Technologies, College of Chemistry & Chemical Engineering, Yan’an University, Yan’an, 716000 People’s Republic of China; 3 School of Chemical Engineering, Guizhou Minzu University, Guiyang, 550025 People’s Republic of China; 4 Analysis Technology R&D Center, Beijing University of Chemical Technology, Beijing, 100029 People’s Republic of China

**Keywords:** Li-rich layered cathodes, Anionic redox reaction, Surface tailoring, CEI layer

## Abstract

In the ongoing pursuit of energy-dense lithium-ion batteries (LIBs), Li-rich layered oxide (LLO) cathodes provide an attractive path forward with extraordinary capacity delivery and low raw material cost. However, the surface-originated degradations and local structure rearrangement incur severe capacity and voltage fading, which fundamentally impedes the practical deployment of LLOs. Here, we report a pre-constructed multifunctional carbon/fluorine-rich artificial cathode electrolyte interface (CEI) layer via a facile thermal treatment. This surface integrated layer, with high-voltage tolerance and amorphous features, is designed to mitigate surface-originated parasitic reactions and phase transformations induced by electrochemistry. Multiscale characterizations reveal that the artificial CEI layer exhibits excellent interfacial compatibility with both the LLO cathode and the electrolyte system, ensuring highly reversible anionic redox and low activation barrier for Li^+^ transport. Profiting from this architecture, remarkable cycling stability is achieved including 90.6% retention after 300 cycles in half cell, along with high energy efficiency and markedly alleviated voltage fading. More importantly, the artificial CEI layer demonstrates a distinct depolarization effect, which almost completely prevents the rise in charging voltage upon cycling. This work provides valuable insights into surficial/interfacial modulations and broadens research directions for designing high-voltage cathodes with more durable interphases for next-generation batteries.

**Supplementary Information:**

The online version contains supplementary material available at 10.1007/s40820-026-02276-8.

## Highlights

- A multifunctional carbon/fluorine-rich artificial cathode electrolyte interphase (CEI) layer is fabricated on Li_1.2_Ni_0.2_Mn_0.6_O_2_ cathode surface.
- The artificial CEI layer ensures highly reversible anionic redox, with higher Coulombic efficiency, suppressed local structure rearrangement, and lowered Li^+^ transport activation barrier.
- Profiting from the mitigated surface-originated degradations, capacity retention up to 90.6% after 300 cycles is achieved, with markedly alleviated voltage decay and effective surface depolarization.

**Supplementary Information:**

The online version contains supplementary material available at 10.1007/s40820-026-02276-8.

## Graphical abstract

**Figure:**
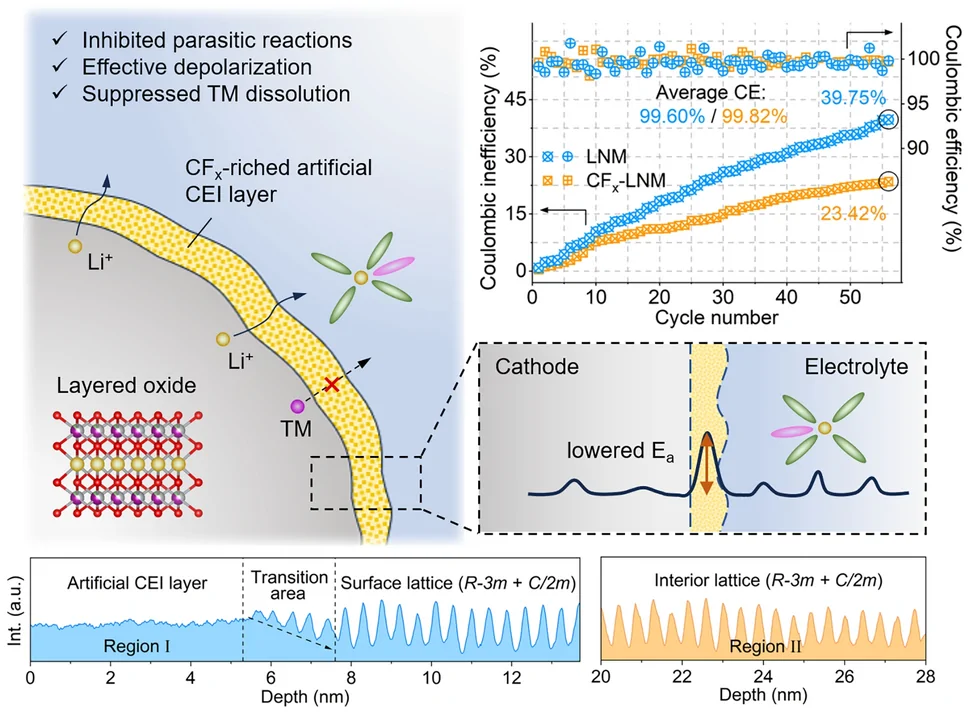

## Introduction

Lithium-ion batteries have emerged as the dominant energy storage solution, serving as the ubiquitous power source for a diverse range of applications including portable electronics, industrial power tools, and the rapidly expanding electric vehicle market. The relentless pursuit of higher energy density has spurred extensive efforts in the research and development of advanced high-specific-energy cathode materials. Lithium-rich layered oxides (LLOs), with reversible capacity exceeding 250 mAh g^−1^, are regarded as one of the most competitive cathode candidates for overcoming the current energy density bottleneck [1–4]. The remarkable capacity of LLOs stems from the strategic utilization of hybrid anion- and cation-redox (HACR) chemistry. Nevertheless, the heavy utilization of anion redox inevitably compromises the solid oxide framework, resulting in the formation of O–O dimers and, ultimately, the release of molecular O_2_ from the particle surface. This phenomenon sets the stage for cathode degradation, as studies indicate that once oxygen vacancies are generated at the LLO particle surface, they could readily propagate toward the particle core and detriment the oxide lattice [5–8]. The following degradations, including local structural rearrangement from layered to disordered phases, transition metal (TM) migration and TM dissolution, eventually give rise to continuous capacity and voltage fading. The emission of O_2_ into the electrolyte also accelerates decomposition of the latter, leading to the formation of a loose and thick cathode electrolyte interface (CEI) with increased surface polarization [9–11]. In this context, the cathode surface/interface plays a pivotal role in the electrode process and its reliability can deeply influence the electrochemical performances of LLOs.

To tackle this, surface engineering strategies such as surface doping/coating, partial lattice reconstruction from layered to spinel/rocksalt-like phases, and defect inducement on surface cation/anion sites, have been widely explored [5, 12–20]. Admittedly those efforts have made extensive contributions toward cathode performance enhancement, most of them aim at reinforcing the cathode surface lattice while neglecting the interfacial issues upon organic electrolyte/Li salt attaching, throwing obstacles in the way of direct CEI stabilization and sustained interfacial protection during prolonged cycling.

Typically, the structures and properties of CEI layer are dictated by the electrolyte system when cycled within a certain potential range. The full utilization of anion redox necessitates an upper cut-off voltage *U*^up^ of approximately 4.8 V, which surpasses the oxidative stability limit of conventional carbonate electrolytes (e.g., approximately 4.5 V for ethylene carbonate, EC), and thus triggers electrolyte decomposition and formation of CEI layer [21–23]. Since this layer forms in situ during cycling, the most applied CEI modification strategies are modulating of electrolyte component/additives, while its direct manipulation presents significant difficulties. An ideal CEI for LLOs should possess high chemical/electrochemical stability against oxidation at *U*^up^, low interface polarization, and good interfacial compatibility. Yet this presents challenges for electrolyte design since the ones should also balance the character of solid electrolyte interface (SEI) on the anode side, as well as other physical properties for ion transport and solvation chemistry [24].

In this work, we present a pre-constructed artificial CEI strategy on a Li-rich layered cathode Li_1.2_Ni_0.2_Mn_0.6_O_2_. The controlled thermal treatment of LLO with 1,1,2,2-tetrafluoroethyl 2,2,3,3-tetrafluoropropyl ether (TTE) enables the formation of a carbon- and fluorine-rich surface layer on the cathode particle. This layer possesses an amorphous feature and is tightly integrated on the cathode surface with a thickness of ~ 6 nm. Proof-of-concept tests indicate that when cycled in an electrolyte non-oxidated environment (against a Li_4_Ti_5_O_12_ anode), this layer could markedly enhance electrode reversibility with higher Coulombic efficiency (CE) and less irreversible charge transfer. Various characterization techniques further demonstrate that this artificial CEI layer significantly mitigates surface-initiated parasitic reactions, alleviates phase transformations and TM dissolution. More importantly, the tailored interfacial chemistry also leads to pronounced depolarization. Leveraging these merits, remarkable cycling stability is achieved including a capacity retention of 90.6% after 300 cycles with less voltage attenuation, alongside improved full cell performance. This work provides actionable insights into surface/interface modulation and opens new avenues for designing high-voltage cathodes for next-generation batteries.

## Experimental Section

### Materials

Lithium acetate dihydrate (CH_3_COOLi·2H_2_O, 99.0%), nickel acetate tetrahydrate (Ni(CH_3_COO)_2_·4H_2_O, 99.0%), manganese acetate tetrahydrate (Mn(CH_3_COO)_2_·4H_2_O, 99.0%), and N-methylpyrrolidone (NMP, 99.5%) were purchased from Shanghai Aladdin Biochemical Technology Co., Ltd. Citric acid (C_6_H_8_O_7_, 99.5%) and ammonia solution (NH_3_·H_2_O, 25%-28%) were purchased from Shanghai Macklin Biochemical Co., Ltd. Polyvinylidene fluoride (PVDF) and Super P were sourced from Canrd Technology Co. Ltd. CR-2025 coin cell cases were purchased from Hefei Kejing Materials Technology Co., Ltd. The 1.0 M LiPF_6_ in EC:EMC:DMC = 1:1:1 wt% electrolyte and 1.0 M LiClO_4_ in EC:EMC:DMC = 1:1:1 vol% electrolyte were purchased from Suzhou Duoduo Chemical Technology Co., Ltd. All chemicals were used directly without any purification.

### Materials Synthesis

#### Preparations of LNM

The Co-free Li_1.2_Ni_0.2_Mn_0.6_O_2_ (LNM) cathode was prepared using a conventional sol–gel (S-G) method combined with solid-state reaction. In a typical synthesis, stoichiometric quantities of CH_3_COOLi·2H_2_O (5% excess to compensate for lithium volatilization during calcination), Ni(CH_3_COO)_2_·4H_2_O, and Mn(CH_3_COO)_2_·4H_2_O were first dissolved thoroughly in deionized water. This transparent solution was then added dropwise into a citric acid solution (molar ratio of citric acid to total metal ions = 1:1) under constant stirring. The pH of the resulting mixture was carefully adjusted to 6.5–7 by dropwise addition of dilute aqueous ammonia. Maintaining this near-neutral pH ensures full deprotonation of citric acid and strong chelation with metal ions, forming a homogeneous and stable sol without any precipitation. After stirring at 80 °C for 8 h, this gel was dried in an air oven at 220 °C overnight to completely decompose the citric acid, yielding a black, porous precursor. The dried precursor was ground, pre-calcined at 450 °C in air for 5 h (heating rate of 5 °C min⁻^1^), and finally annealed at 900 °C for 20 h to obtain the well-crystallized layered LNM oxide.

#### Fabrication of Artificial CEI Layer

To fabricate a carbon- and fluorine-rich artificial CEI layer on the LNM cathode, 1,1,2,2-tetrafluoroethyl 2,2,3,3-tetrafluoropropyl ether (TTE, purchased from Suzhou Duoduo Chemical Technology Co., Ltd. and used as received without further purification) was adopted as the precursor. After calcination, 2 g of the as-obtained LNM powder was quenched into 20 mL of TTE and then subjected to intense ultrasonication for 20 min. The resulting dispersion was transferred into a 50-mL Teflon-lined hydrothermal reactor and heated at 220–320 °C for 6–12 h. After the thermal treatment, the product was collected and dried in a vacuum oven at 90 °C for 10 h to thoroughly remove residual TTE, yielding cathode with an artificial CEI layer. After a series of trial-and-error experiments, the reaction temperature was optimized to 270 °C and the reaction time to 6 h (Fig. S1 and Table S1). The optimized cathode was marked as CF_x_-LNM.

## Results and Discussion

**Fig. 1:**
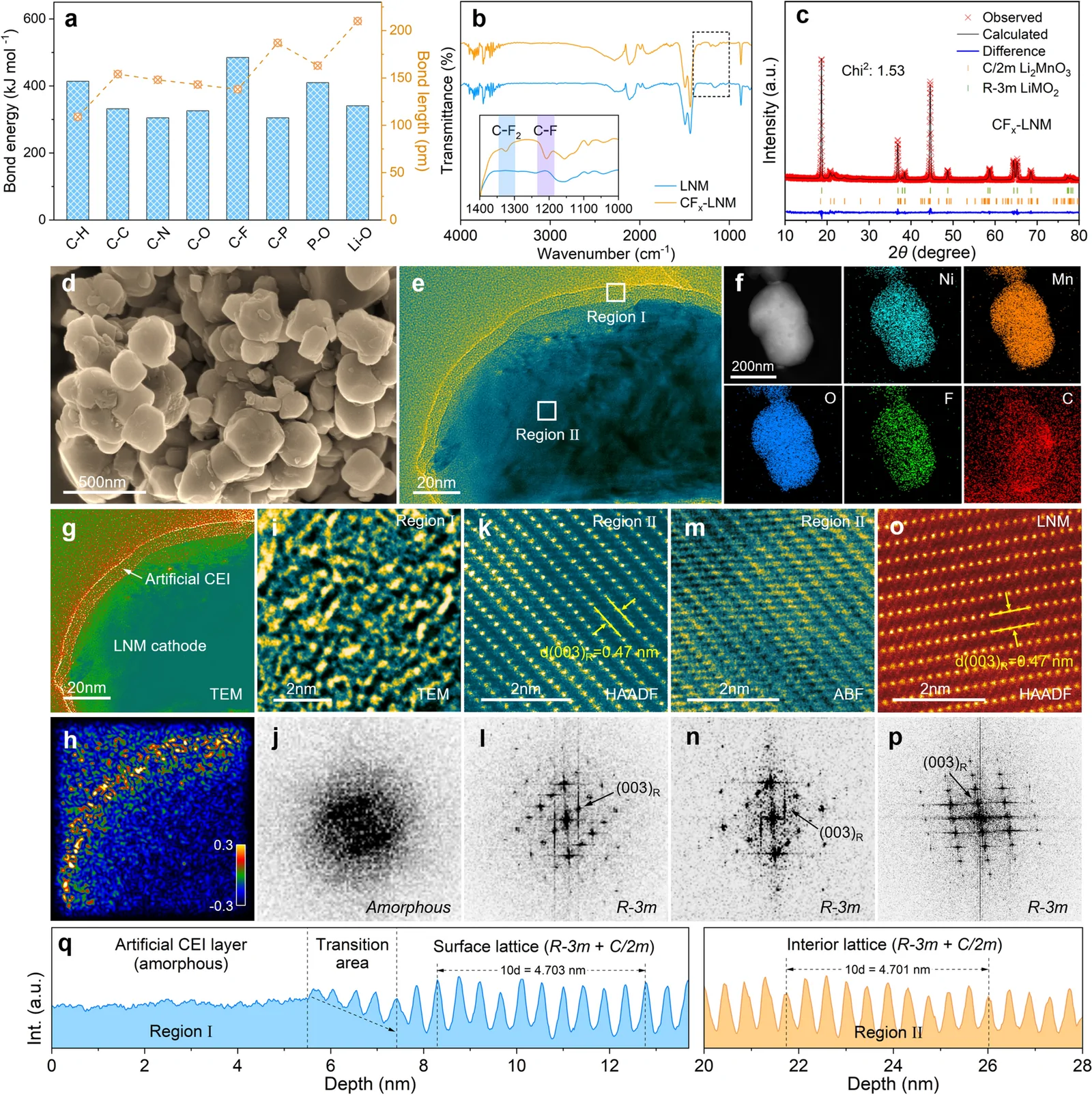
Morphological and structural characterizations of LNM and CF_x_-LNM. **a** Comparison of bond energy and bond length of single-bonded functional groups potentially suitable for artificial CEI layer. **b** FT-IR spectra of LNM and CF_x_-LNM. **c** XRD pattern and Rietveld refinement result of CF_x_-LNM. **d** SEM and **e** TEM images of CF_x_-LNM. **f** EDS elemental mapping results of CF_x_-LNM, showing the distribution of Ni, Mn, O, F, and C. **g** TEM image and **h** GPA results of CF_x_-LNM, homologous with **e**. **i-n** Magnified HAADF-STEM images and corresponding FFT patterns for different regions of CF_x_-LNM. **o, p** Magnified HAADF-STEM image and corresponding FFT pattern of LNM. **q** STEM line scan profile of CF_x_-LNM, illustrating the near-surface atomic arrangement

Our investigation commenced with the screening of single-bonded functional groups potentially suitable for an artificial CEI layer. Given that chemical/electrochemical stability against high-voltage oxidation is the critical determinant for LLOs, bond energy and length were assessed as the key parameters in this assessment (Fig. 1a). Of the candidates considered, the C-F component demonstrates the highest bond energy (approximately 485 kJ mol^−1^) together with a moderate bond length. TTE was therefore chosen as the raw reagent, owing to its high content of -CF/-CF_2_ groups and the presence of a relatively labile ether linkage that readily cleaves during thermal processing. The detailed experimental procedures are articulated in the Materials Synthesis section, and the formation mechanism and ion transport behavior in the artificial CEI are discussed in Supplementary Notes and Discussions. For conciseness, the pristine Li_1.2_Ni_0.2_Mn_0.6_O_2_ is denoted as LNM, while the cathode with the pre-constructed artificial CEI layer is denoted as CF_x_-LNM hereafter. Fig.  1b illustrates the Fourier transform-infrared (FT-IR) spectra of LNM and CF_x_-LNM. Both cathodes exhibit typical absorption peaks corresponding to LLOs, while two extra peaks emerge in CF_x_-LNM. These are ascribed to the presence of -CF (around 1180–1240 cm^−1^) and -CF_2_ (around 1300–1350 cm^−1^) groups, respectively [25, 26]. The absence of characteristic ether bond vibrations (around 900–1100 cm^−1^) indicates that the -CF_x_ signature originates from fluoro-ether decompositions derivatives, rather than a simple TTE physical adsorption (Fig. S2). Figures  1c and S3 present the X-ray diffraction (XRD) patterns of LNM and CF_x_-LNM. All diffraction peaks can be well indexed to LLOs, which comprises a combination of hexagonal LiMO_2_ with *R* $\overline{3 }$ *m* space group and monoclinic Li_2_MnO_3_ with *C2/m* space group [20, 27, 28]. Rietveld refinement confirms that the artificial CEI layer and its construction process did not alter the bulk crystal structure, as evidenced by the negligible difference in lattice parameters between the two cathodes (Tables S2 and S3).

Scanning electron microscopy (SEM) and high-resolution transmission electron microscopy (HRTEM) were employed to characterize the morphology of LNM and CF_x_-LNM. As shown in Figs. 1d and S4, both cathodes consist of granular particles with diameters of 200–400 nm. HRTEM image clearly shows that a continuous and uniform layer tightly integrated on the CF_x_-LNM surface (Figs. 1e and S5). The homogeneous distribution of all elements (Ni, Mn, O for LNM and Ni, Mn, O, F, C for CF_x_-LNM) was further verified by HRTEM-equipped energy-dispersive spectrometer (EDS) as shown in Figs. 1f and S6 and Table S4. The obvious TEM contrast discrepancy (Fig. 1g) and geometric difference (according to geometric phase analysis, GPA, Fig. 1h) indicate a clear structure distinction between the surface layer and oxide bulk. Figure 1i–n illustrates the magnified TEM, high-angle annular dark field (HAADF) and annular bright field (ABF) images, along with homologous fast Fourier transform (FFT) patterns for region I and II in Fig. 1e. The artificial CEI layer possesses a typical amorphous feature according to Fig. 1i, j, which is anticipated to facilitate ion transport during electrochemistry. For the interior region, atomic-resolution HAADF and ABF images resolve a well-ordered O3-type layered structure with negligible signal of Li/Ni antisite defects. This bulk structure is consistent with that of the pristine LNM cathode (Fig. 1o, p). Accordingly, the STEM line scan results in Fig. 1q reveal the near-surface architecture of CF_x_-LNM, comprising an ~ 6 nm amorphous surface layer, a transition region, and an ordered interior lattice with an interplanar spacing of ~ 0.47 nm.

**Fig. 2:**
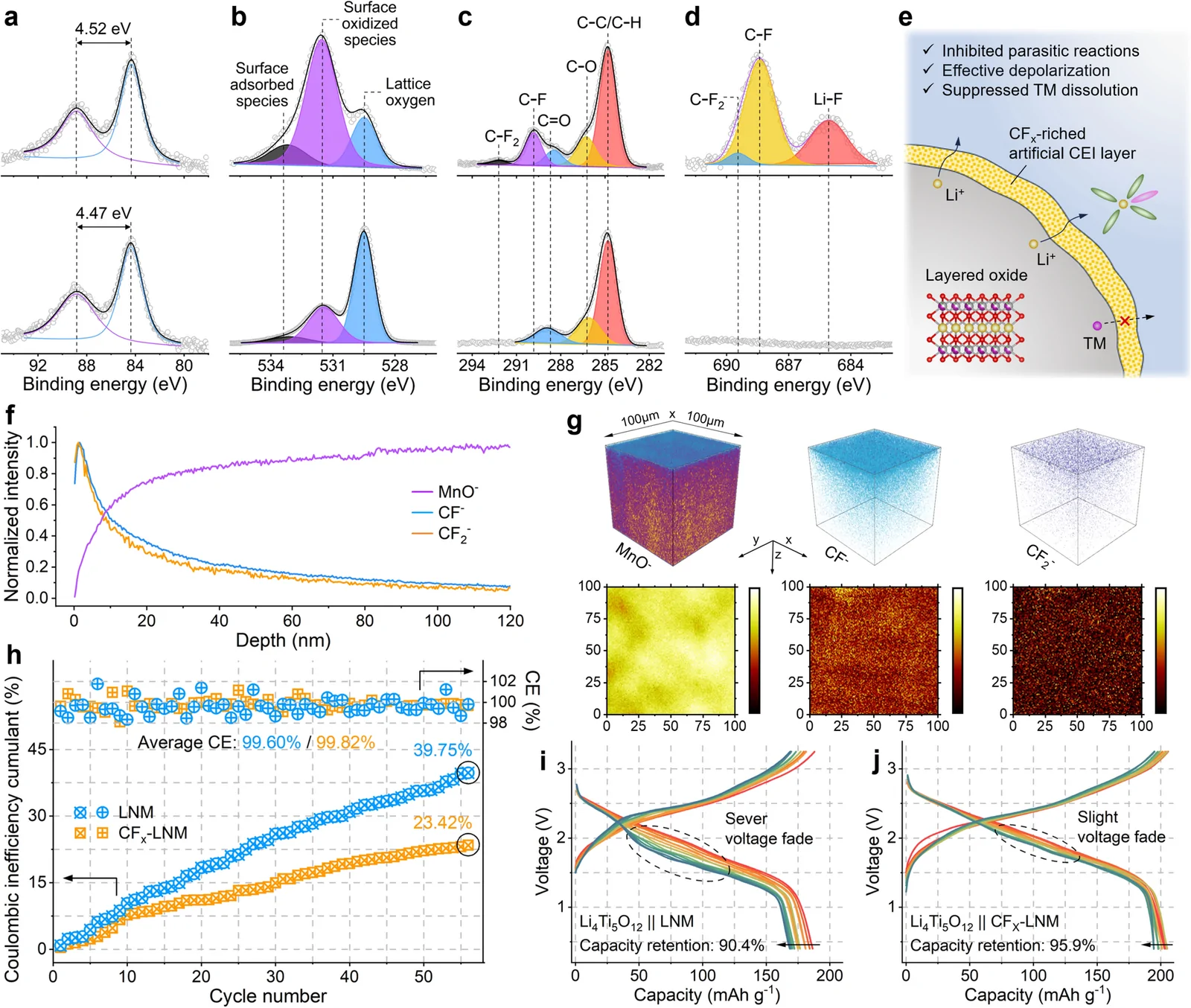
Chemical composition and properties of the artificial CEI layer. XPS spectra of **a** Mn 3*s*, **b** O 1*s*, **c** C 1*s*, and **d** F 1*s* for LNM and CF_x_-LNM. **e** Schematic diagram of CF_x_-LNM, showing the structure and function of the artificial CEI layer. **f** TOF–SIMS depth profiles of CF^−^, CF_2_^−^, and MnO^−^ fragments. **g** 3D reconstruction and corresponding 2D top views of the representative fragments. **h** Coulombic inefficiency cumulant and Coulombic efficiency for LNM and CF_x_-LNM in cells with a Li_4_Ti_5_O_12_ counter electrode. Galvanostatic charge/discharge curves of **i** Li_4_Ti_5_O_12_||LNM and **j** Li_4_Ti_5_O_12_||CF_x_-LNM full cell

Surface chemistry was subsequently investigated by X-ray photoelectron spectroscopy (XPS). It is found that the artificial CEI did not alter the Mn oxidation state, as shown by the nearly identical Mn 3*s* splitting energies between the two cathodes in Figs. 2a and S7 [29]. Nevertheless, CF_x_-LNM exhibits significantly weakened lattice oxygen signal alongside an increase in surface oxidized species (Fig. 2b) [30]. Given the high surface sensitivity of XPS, these observations reflect the complete coverage of the artificial layer, which reduces the lattice oxygen contributing to the spectrum. As for the C 1*s* and F 1*s* spectra (Fig. 2c, d), characteristic peaks corresponding to both C-F and C-F_2_ are clearly identified, again implying the chemical constituents of the surface layer [25, 31]. To obtain further statistical information of the surface structure, time-of-flight secondary ion mass spectrometry (TOF–SIMS) was carried out. Figure 2f depicts the normalized intensity of representative fragments as a function of sputtering time. Both CF^−^ and CF_2_^−^ fragments exhibit a depth-dependent distribution, of which the intensities reach a maximum rapidly and subsequently turn to decline. In contrast, the MnO^−^ signal shows a progressive increase during the initial 100 s of sputtering before reaching a nearly steady state. Figure 2g presents the three-dimensional (3D) reconstruction and two-dimensional (2D) top view results of the fragments. The uniform in-plane signals of CF^−^ and CF_2_^−^ fragments in the 2D maps confirm the homogeneity of the surface layer, while the 3D renders indicate the gradient spatial distribution. These results further elucidate the structure and composition of the as-constructed CEI, in well line with the FT-IR and HAADF-STEM findings.

To evaluate the effect of the artificial CEI during electrochemical process, a proof-of-concept test was conducted by operating the cathodes within an electrolyte non-oxidizing environment. A Li_4_Ti_5_O_12_ counter electrode was adopted due to its moderate (de)lithiation plateau at ~ 1.55 V, which results in a ~ 2 V voltage gap between the electrodes (corresponding to a voltage range of 0.4–3.25 V) and thus avoids CEI formation through carbonate electrolyte oxidation (Fig. S8). When cycled within a certain voltage range, the Coulombic efficiency (CE) is defined as the ratio of the total reductive charge (*Q*_red_) to the oxidative charge (*Q*_oxi_) [32]:

$${\mathrm{C}\mathrm{E}}_{n}=\frac{{Q}_{\mathrm{r}\mathrm{e}\mathrm{d}}(n; {U}_{\mathrm{m}\mathrm{i}\mathrm{n}},{U}_{\mathrm{m}\mathrm{a}\mathrm{x}})}{{Q}_{\mathrm{o}\mathrm{x}\mathrm{i}}(n; {U}_{\mathrm{m}\mathrm{i}\mathrm{n}},{U}_{\mathrm{m}\mathrm{a}\mathrm{x}})}$$

The irreversible charge transfer during electrochemistry is then able to be quantified by the Coulombic inefficiency cumulant (CIC), i.e., the accumulation of the Coulombic efficiency loss in each individual cycle:

$${\mathrm{C}\mathrm{I}\mathrm{C}}_{n}=\sum_{{n}^{^{\prime}}=m}^{n}|1-{\mathrm{C}\mathrm{E}}_{{n}^{^{\prime}}}|$$

Figure 2h plots the CIC and average CE for both cathodes after an initial activation cycle. It is evident that the artificial CEI layer effectively mitigates the CIC increase, lowering it to 23.42% for CF_x_-LNM from 39.75% for the pristine LNM. Meanwhile, the average CE improves from 99.60% (for LNM) to 99.82% (for CF_x_-LNM), indicating a more reversible electrochemistry for the latter. The detailed charge–discharge profiles are provided in Figs. 2i, j and S9. The slight voltage fade and a capacity retention up to 95.9% for CF_x_-LNM again imply the efficacy of the artificial CEI layer on cathode stabilization. As such, the properties and functions of the artificial CEI layer are summarized in Fig. 2e. To better assess the lattice oxygen stability and more directly monitor the gas evolution behavior, differential electrochemical mass spectrometry (DEMS) measurements were also conducted during the initial charging process. As shown in Fig. S10, substantial O_2_ evolution is observed from LNM near the end of the first charge, with a peak concentration reaching 200 ppm. In contrast, O_2_ evolution from CF_x_-LNM is significantly suppressed, and only a very minor amount of O_2_ is detected throughout the entire charging process. These results provide direct evidence for the suppression of O_2_ evolution by the artificial CEI layer.

**Fig. 3:**
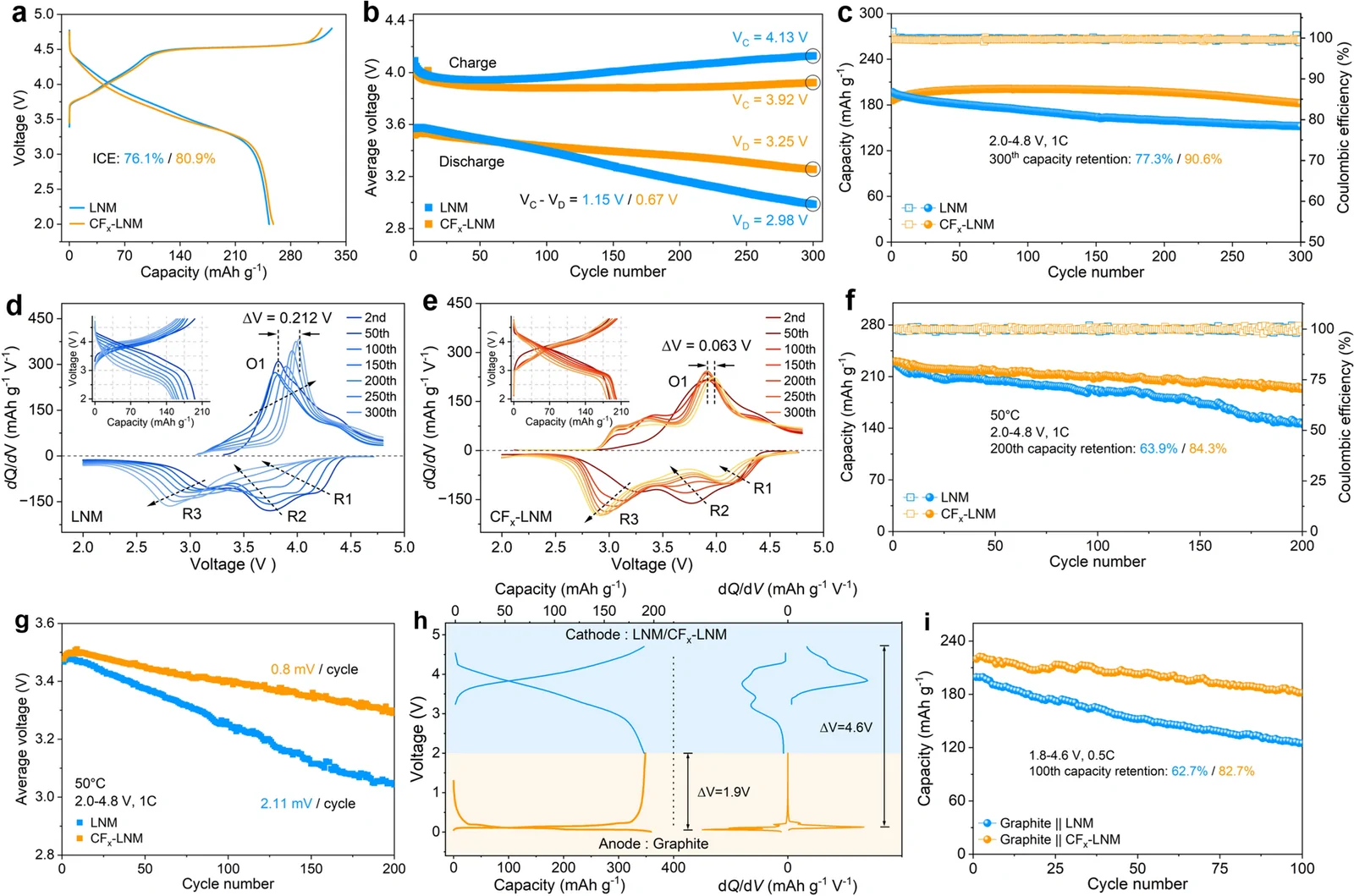
Electrochemical behaviors of LNM and CF_x_-LNM in half and full cells. **a** Initial charge/discharge profiles of LNM and CF_x_-LNM at 0.1 C (1 C ≡ 250 mAh g^−1^). Cycle number as a function of **b** average charge/discharge voltage and **c** discharge capacity at 1 C. Selected dQ/dV curves of **d** LNM and **e** CF_x_-LNM over 300 cycles. Insert, corresponding charge/discharge profiles. Cycle number as a function of **f** discharge capacity and **g** average discharge voltage at 50 °C in half cells. **h** Schematic diagram of the Graphite||LNM and Graphite||CF_x_-LNM full cells. **i** Cycling performance of the full cells

The half cell performances were evaluated with Li metal anodes in a voltage range of 2.0—4.8 V. CF_x_-LNM exhibits a higher initial CE of 80.9% (vs. 76.1% for LNM) with a shortened oxygen oxidation plateau (Fig. 3a). When cycled at 1 C (Figs. 3b–e and S11 and Table S5), significantly improved cyclability can be observed in CF_x_-LNM with capacity retention of 90.6% (vs. 77.3% for LNM) and higher energy efficiency within 300 cycles. More importantly, the artificial CEI layer demonstrates a marked depolarization effect, preserving CF_x_-LNM with an average discharge voltage V_D_ of 3.25 V at the 300th cycle while keeping the average charge voltage V_C_ nearly constant at 3.92 V throughout cycling. This is in sharp contrast with LNM, the V_D_ of which at the 300th cycle deteriorates to 2.98 V and V_C_ rises to 4.13 V. The redox couple variations during cycling are also studied by the differential capacity (*d*Q/*d*V) analysis (Fig. 3d, e). The negligible O1 peak changes (ΔV = 0.063 V for CF_x_-LNM vs. ΔV = 0.212 V for LNM) and the mitigated R1-3 peak variations indicate a more reversible redox chemistry enabled by the artificial CEI. This is further evidenced by the cyclic voltammetry tests after 4 and 99 cycles in a three-electrode cell (Fig. S12). The stabilizing effect persists even at an elevated temperature of 50 °C, under which the cathode degradation and surface/interface parasitic reactions are significantly intensified (Figs. 3f, g and S13). As a result, an improved capacity retention of 84.3% (vs. 63.9% for LNM) alongside a substantially mitigated voltage fading rate of 0.80 mV/cycle (vs. 2.11 mV/cycle for LNM) were achieved on CF_x_-LNM.

To better assess the application potential of the cathode with artificial CEI layer, full cells were assembled employing a commercial graphite anode (Figs. 3h and S14). A voltage range of 1.8–4.6 V and current density of 0.5 C were adopted. As expected, the full cell with CF_x_-LNM cathode exhibits a more stable redox behavior with a capacity retention of 82.7% after 100 cycles. These findings substantiate that the artificial CEI layer significantly enhances the overall performance of Li-rich cathode, particularly by mitigating irreversible capacity/voltage losses and enabling effective surface depolarization.

**Fig. 4:**
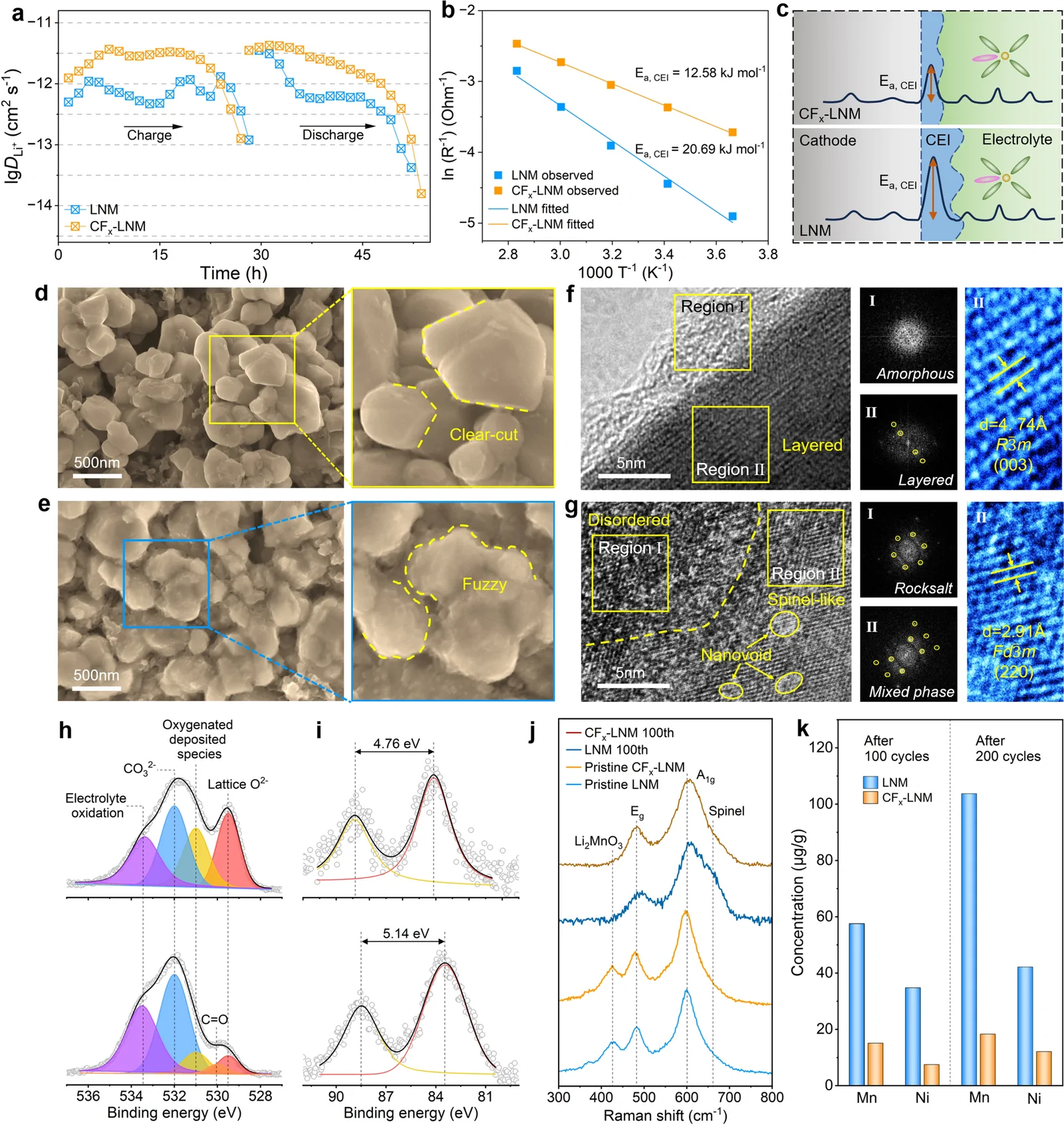
Kinetics and post-mortem analyses of the cathodes after cycling. **a** Li^+^ diffusion coefficient of LNM and CF_x_-LNM after 100 cycles, calculated based on the GITT results. **b** Comparison of activation energies for Li^+^ migration across the CEI layer in LNM and CF_x_-LNM half cells. **c** Schematic diagram of the Li^+^ transport kinetics through the CEI layer for LNM (bottom) and CF_x_-LNM (top) half cells. SEM images of **d** CF_x_-LNM and **e** LNM after 100 cycles. HRTEM images and corresponding FFT patterns of **f** CF_x_-LNM and **g** LNM after 100 cycles. XPS spectra of **h** O 1 *s*, **i** Mn 3*s* for the cathodes after 100 cycles. **j** Raman spectra of LNM and CF_x_-LNM before and after cycling. **k** ICP-OES results, showing the dissolution amounts of Ni and Mn ions after different cycles

The mechanism underlying cathode performance improvement by the artificial CEI layer was subsequently investigated through post-cycling analysis. We first employed the galvanostatic intermittent titration technique (GITT) to probe the Li^+^ diffusion kinetics after 100 cycles. It is found that CF_x_-LNM demonstrates a higher Li^+^ diffusion coefficient (*D*_Li+_) than LNM across every measured state of charge (Figs. 4a and S15). Temperature-dependent electrochemical impedance spectroscopy (EIS) was carried out to investigate the activation energy (E_a_) barrier for Li ion pre-desolvation and its transport through the CEI layer [33]. As plotted in Figs. 4b, c and S16 and Table S6, the *E*_a,CEI_ and *E*_a,ct_ are 12.58 and 15.46 kJ mol^−1^, respectively, which are much lower than those (20.69 kJ mol^−1^ for *E*_a,CEI_ and 17.14 kJ mol^−1^ for *E*_a,ct_) of LNM. This lower energy barrier accounts for the observed kinetic enhancement and the increased *D*_Li+_ in CF_x_-LNM compared to the unmodified cathode.

SEM and HRTEM were performed on the cathodes extracted from post-cycled electrodes. CF_x_-LNM surface remains clean and smooth according to the SEM images (Fig. 4d), showing little morphological change compared with its pristine state. In sharp contrast, LNM surface is covered by a thick and fuzzy layer, as a consequence of severe electrolyte oxidization and deposition (Fig. 4e). The elimination of these electrochemical by-products plays an important role in the surface depolarization and kinetic improvement of CF_x_-LNM. HRTEM images and their corresponding FFT patterns further reveal that CF_x_-LNM possesses a uniform surface layer with amorphous features and a well-preserved interior layered structure with *d*_(003)_ spacing of 0.474 nm (Fig. 4f). However, LNM exhibits a defective surface with partially disordered and spinel-like phases, as well as nanovoids induced by electrolyte corrosion (Fig. 4g). Specifically, a spinel character with *d*_(220)_ spacing of 0.291 nm (*Fd* $\overline{3 }$ *m* symmetry) is clearly observed, indicating an extensive local cation mixing and lattice deterioration.

The surface chemistry of the cycled cathodes was explored by XPS. As shown in Figs. 4h and S17, the O 1*s* spectra can be attributed to five peaks: the electrolyte oxidation products, carbonate species (CO_3_^2−^), oxygenated deposited species, C=O components, and lattice O^2−^, respectively [34–38]. After 100 cycles, the lattice O^2−^ signal of LNM is significantly diminished, while the peaks corresponding to electrolyte oxidation products and CO_3_^2−^ become more intense. These observations indicate that severe parasitic reactions have occurred, which trigger the carbonate electrolyte oxidation and decomposition. By contrast, CF_x_-LNM shows a cleaner surface, as evidenced by the more intense lattice O^2−^ signal and the absence of C=O peak. In the Mn 3*s* spectra, a higher Mn valence is observed in CF_x_-LNM (Fig. 4i), with averaged oxidation state (AOS) of + 3.60 (vs. + 3.17 for LNM). The preservation of Mn valence gives rise to mitigated Jahn–Teller distortion and less spinel-like structure evolution [39]. The improved surface structure integrity and mitigated parasitic reactions result in less charge-transfer resistant growth and better kinetics, as evidenced by the EIS results (Fig. S18). In the Raman spectra (Fig. 4j), both cathodes exhibit the disappearance of Li_2_MnO_3_ signals after cycling, as a consequence of complete activation of anionic redox. In addition to the vibration signals of the layered E_g_ and A_1g_ modes, a new peak with spinel characters (located at ~ 660 cm^−1^) emerges only in LNM, in well line with the HRTEM observation [40, 41]. ICP-OES results show that the dissolution of Mn and Ni is significantly suppressed in CF_x_-LNM across different cycles (Figs. 4k and S19), suggesting that the engineered artificial CEI layer effectively preserves lattice integrity and mitigates electrolyte corrosion.

**Fig. 5:**
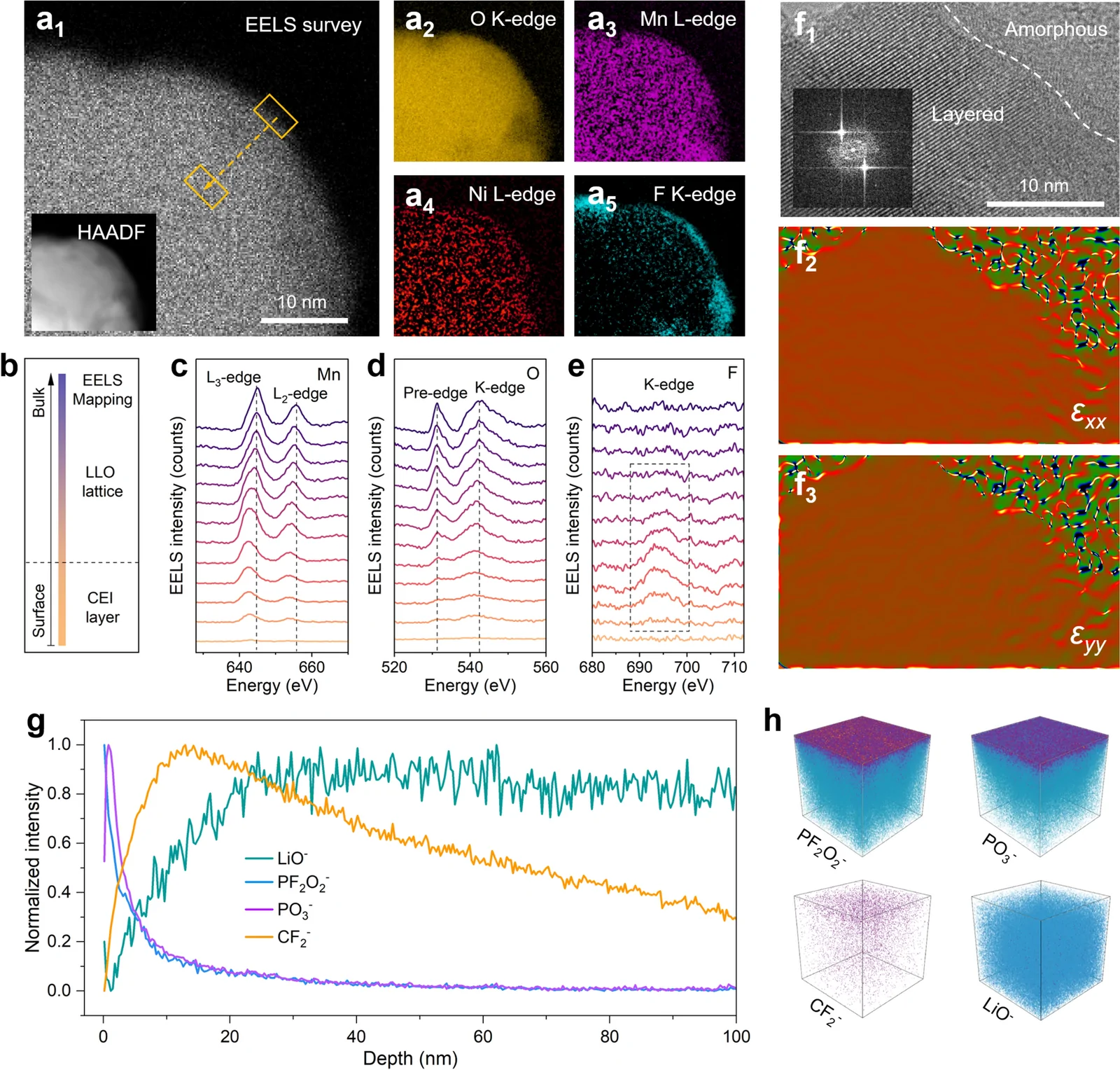
Evolution of the CEI and variation of surface chemical species after electrochemical cycling. **a** EELS mapping results of a representative cycled CF_x_-LNM particle, showing the distribution of O, Mn, Ni, and F elements. **b-e** Detailed Mn, O, and F spectra at different particle depths, extracted from EELS mapping result. The ROI is marked in **a** by yellow rectangular. **f** HRTEM and GPA results for post-cycled CF_x_-LNM. Inset, the corresponding FFT pattern. **g** TOF–SIMS results, showing the normalized intensity of LiO^−^, PF_2_O_2_^−^, PO_3_^−^, and CF_2_^−^ fragments as a function of sputtering depth. **h** 3D reconstruction results of the fragments

To gain a deeper insight into the evolution of CEI and variation of surface chemical species during electrochemistry, electron energy loss spectroscopy (EELS) was conducted on CF_x_-LNM after 50 galvanostatic cycles. In this section, half cells were assembled using a LiClO_4_-based electrolyte (1.0 M LiClO_4_ in EC:EMC:DMC = 1:1:1 vol%) and a lithiated polyacrylic acid (PAALi) binder to avoid extraneous fluorine contamination. As illustrated in Fig. 5a, the F K-edge signal is clearly identified with a surface-enriched character, whereas O, Mn, and Ni signals exhibit a homogeneous distribution across the entire particle. This confirms that the artificial CEI layer is well-retained after extended cycling and still sustains its surface-integrated morphology. Figure 5b–e presents the detailed EELS spectra extracted from a rectangular region of interest (ROI) spanning from the particle surface to the interior. A slight shift of the Mn L_2,3_-edges is discernible, attributed to the electroactivated Mn^3+/4+^ redox couple and partial Mn dissolution, consistent with the XPS and ICP results. The O pre-edge lineshape is well retained, signifying the integrity of the near-surface oxide lattice with markedly diminished oxygen loss and phase rearrangement. The F signal is detectable only within the outermost few nanometers, directly attesting to the enduring presence of the artificial CEI layer. It should be noted that while the use of a fluorine-free electrolyte was necessary to unambiguously resolve the artificial CEI signals, the structural protection against surface oxygen loss conferred by this layer is an intrinsic characteristic that remains operative irrespective of the electrolyte anion chemistry.

The structural integrity of the post-cycled cathodes was further assessed by HRTEM and GPA (Figs. 5f and S20). CF_x_-LNM retains well-defined lattice fringes with negligible strain build-up, in stark contrast to LNM, the lattice of which shows pronounced strain accumulation. TOF–SIMS analysis was subsequently conducted to investigate the surface chemical speciation after cycling. The outermost surface of CF_x_-LNM contains most PF_2_O_2_^−^ and PO_3_^−^ fragments, as a consequence of carbonate electrolyte oxidation (Fig. 5g). In contrast, the CF_2_^−^ fragment exhibits a distinct signal assignment, which reaches its maximum and gradually decline. The 3D reconstruction results (Fig. 5h) further substantiate the spatial distribution of these species. Collectively, these results demonstrate that the artificial CEI layer sustains its structural and chemical integrity throughout cycling, affording durable and effective protection to the cathode surface.

**Fig. 6:**
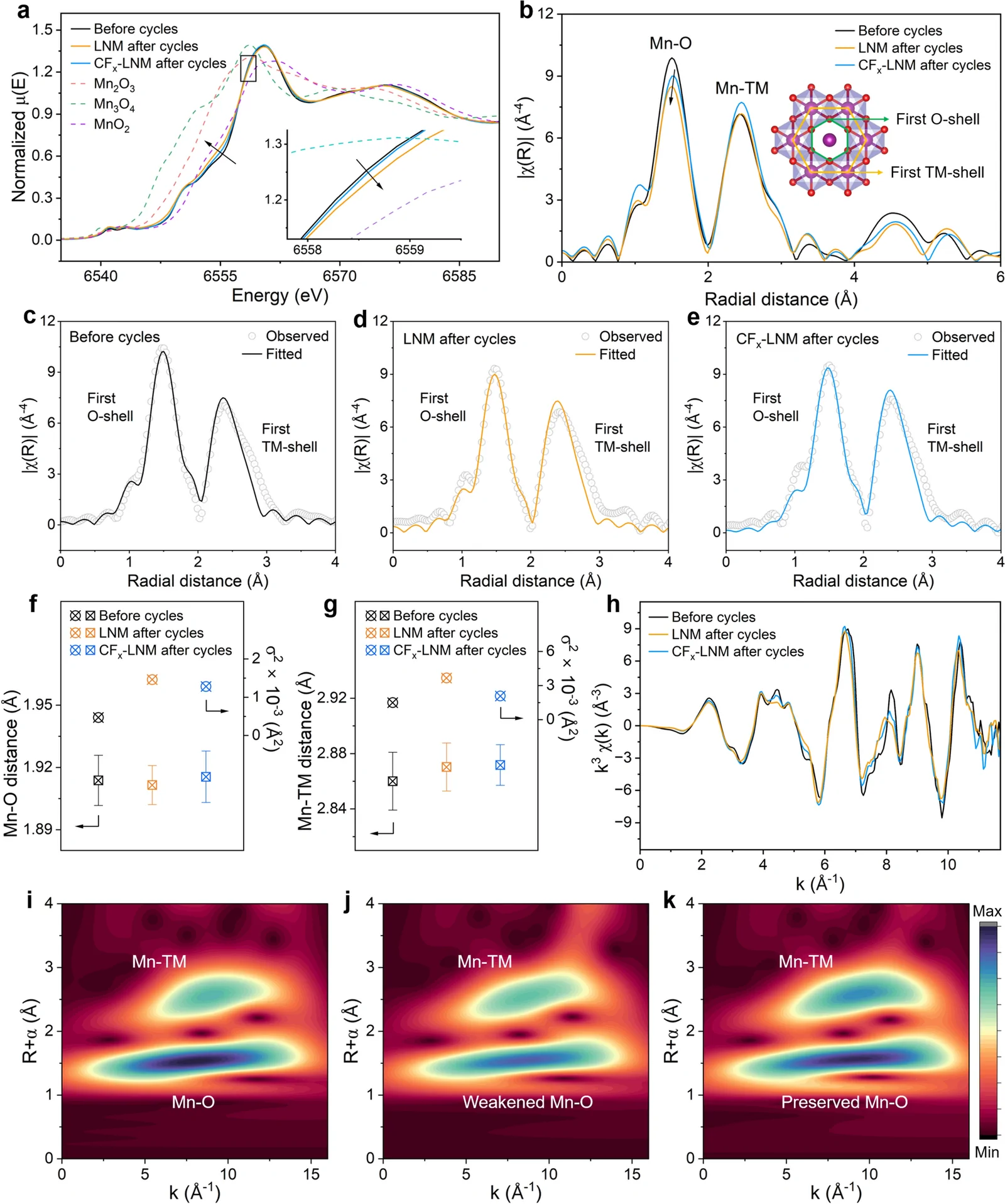
Local structural environment analyses of the post-cycled cathodes by hard X-ray absorption spectroscopy. **a** Normalized Mn K-edge XANES spectra of LNM and CF_x_-LNM after 100 cycles. **b**–**e** Fourier-transformed Mn K-edge EXAFS spectra and corresponding fitting results for LNM and CF_x_-LNM. Fitted Debye–Waller factor and **f** Mn–O bond distance and **g** Mn-TM bond distance for the cathodes. **h**–*k*_3_-weighted XANES spectra. **i**–**k** Wavelet transformed EXAFS results, showing the differences in Mn–O coordination between LNM and CF_x_-LNM

To further reveal the variations on the local coordination environments of LNM and CF_x_-LNM after cycling, hard X-ray absorption spectroscopy (XAS) was carried out. A comparison of the Mn K-edge X-ray absorption near-edge structure (XANES) spectra (Fig. 6a) indicates the difference of Mn oxidation states by the clear divergence in the pre-edge region between LNM and CF_x_-LNM. Figure 6b–e presents the Fourier-transformed extended X-ray absorption fine structure (EXAFS) spectra. The peaks located at approximately 1.51 and 2.45 Å correspond to the Mn–O coordination in the first O shell and the Mn-TM coordination in the first TM shell, respectively [42, 43]. The post-cycled LNM cathode demonstrates a significantly attenuated Mn–O absorption signal, which suggests the formation of oxygen vacancies due to substantial oxygen release during cycling. In contrast, this attenuation is partially alleviated in CF_x_-LNM. Meanwhile, the Mn–O bond distance in LNM contracts slightly, indicating the occurrence of MnO_6_ octahedra distortion; however, such variation is not observed in CF_x_-LNM. The detailed Mn–O/Mn–TM distances and Debye–Waller factor calculated from the fitting results are presented in Fig. 6f, g and Table S7. The minimal changes in σ^2^ compared to the pristine state again demonstrate the better lattice preservation of CF_x_-LNM, which is also corroborated by the *k*^3^-weighted XANES spectra (Fig. 6h) and wavelet transformed EXAFS results (Fig. 6i-k).

## Conclusions

In summary, we propose a pre-constructed multifunctional carbon/fluorine-rich artificial CEI layer on a Li-rich cathode Li_1.2_Ni_0.2_Mn_0.6_O_2_ and showcase its efficacy on stabilizing overall HACR chemistry and on maintaining cathode framework integrity under high-voltage operation. Attributed to its intrinsic stability and well interfacial compatibility on both the cathode and electrolyte sides, this rationally architected layer exhibits a uniform morphology and serves as a robust barrier to protect the surface oxide lattice. The presence of this surface structure enables the LLO cathode to achieve an impressive performance of 90.6% capacity retention over 300 cycles in half cell, and mitigated voltage fading under various operating conditions. Moreover, complementary multiscale characterization techniques demonstrate that the artificial CEI layer sustains its structural and chemical integrity throughout prolonged cycling. Such durability gives rise to a direct depolarization effect and improved surface chemistry, accompanied by a marked suppression of both surface parasitic reactions and transition metal dissolution. This work provides valuable insights into surficial/interfacial modulations and broadens research directions for designing high-voltage cathodes with more durable interphases for next-generation batteries.

## Supplementary Information

Below is the link to the electronic supplementary material.

Supplementary file1 (DOCX 7637 KB)

## References

[1] S. Wang, L. Wang, S. David, T. Liu, C. Zhan et al., Correlating concerted cations with oxygen redox in rechargeable batteries. Chem. Soc. Rev. **53**, 3561–3578 (2024). 10.1039/d3cs00550j 38415295 10.1039/d3cs00550j

[2] S. Jiao, D. Shi et al., Revisiting the structural limitations of layered oxide cathodes for reversible lithium-ion storage. ACS Energy Lett. **11**(2), 1125–1134 (2026). 10.1021/acsenergylett.5c03519

[3] G. Assat, J.-M. Tarascon, Fundamental understanding and practical challenges of anionic redox activity in Li-ion batteries. Nat. Energy **3**(5), 373–386 (2018). 10.1038/s41560-018-0097-0

[4] M. Zheng, X. Zhu, H. Zheng, Z. Bo, J. Lu, Deployment strategies for Li-rich cathode materials in batteries. Nat. Energy **10**(7), 789–792 (2025). 10.1038/s41560-025-01777-x

[5] Z. Xu, X. Guo, X. Zeng, J. Liu, J. Yin et al., Coherent strain-inhibiting phase construction of lithium-rich manganese-based oxide toward high mechanochemical stability. J. Am. Chem. Soc. **147**(5), 3967–3980 (2025). 10.1021/jacs.4c11385 39836949 10.1021/jacs.4c11385

[6] Z. Liu, Y. Zeng, J. Tan, H. Wang, Y. Zhu et al., Revealing the degradation pathways of layered Li-rich oxide cathodes. Nat. Nanotechnol. **19**(12), 1821–1830 (2024). 10.1038/s41565-024-01773-4 39223255 10.1038/s41565-024-01773-4

[7] P. Yan, J. Zheng, Z.-K. Tang, A. Devaraj, G. Chen et al., Injection of oxygen vacancies in the bulk lattice of layered cathodes. Nat. Nanotechnol. **14**(6), 602–608 (2019). 10.1038/s41565-019-0428-8 31011218 10.1038/s41565-019-0428-8

[8] L. Xu, M. Hong, J. Guo, F. Shen, D. Xu et al., Boosting Li^+^ diffusion in lithium-rich oxides through intrinsic structural design: insights and design principles. Nano-Micro Lett. **18**(1), 273 (2026). 10.1007/s40820-026-02099-7 10.1007/s40820-026-02099-7 PMC12963579 41784755

[9] M. Cai, Y. Dong, M. Xie, W. Dong, C. Dong et al., Stalling oxygen evolution in high-voltage cathodes by lanthurization. Nat. Energy **8**(2), 159–168 (2023). 10.1038/s41560-022-01179-3

[10] T. Liu, J. Liu, L. Li, L. Yu, J. Diao et al., Origin of structural degradation in Li-rich layered oxide cathode. Nature **606**(7913), 305–312 (2022). 10.1038/s41586-022-04689-y 35676429 10.1038/s41586-022-04689-y

[11] Y. Dong, J. Li, Oxide cathodes: functions, instabilities, self healing, and degradation mitigations. Chem. Rev. **123**(2), 811–833 (2023). 10.1021/acs.chemrev.2c00251 36398933 10.1021/acs.chemrev.2c00251

[12] Z. Li, Y. Li, M. Zhang, Z.-W. Yin, L. Yin et al., Modifying Li@Mn_6_ superstructure units by Al substitution to enhance the long-cycle performance of co-free Li-rich cathode. Adv. Energy Mater. **11**(37), 2101962 (2021). 10.1002/aenm.202101962

[13] Y. Zhang, Z. Chen, X. Shi, C. Meng, P. Das et al., Regulation of 3d-transition metal interlayered disorder by appropriate lithium depletion for Li-rich layered oxide with remarkably enhanced initial coulombic efficiency and stability. Adv. Energy Mater. **13**(5), 2203045 (2023). 10.1002/aenm.202203045

[14] H. Zhao, W. Li, J. Li, H. Xu, C. Zhang et al., Enhance performances of Co-free Li-rich cathode by eutesctic melting salt treatment. Nano Energy **92**, 106760 (2022). 10.1016/j.nanoen.2021.106760

[15] J. Shen, Y. Lou, J. Sun, H. Li, L. Li et al., Achieving high reversible anionic redox activity of Li-rich layered oxides *via* Mg and Mo co-doping. Adv. Funct. Mater. **35**(36), 2425638 (2025). 10.1002/adfm.202425638

[16] P. Liu, H. Zhang, W. He, T. Xiong, Y. Cheng et al., Lithium deficiencies engineering in Li-rich layered oxide Li_1.098_Mn_0.533_Ni_0.113_Co_0.138_O_2_ for high-stability cathode. J. Am. Chem. Soc. **141**(27), 10876–10882 (2019). 10.1021/jacs.9b04974 31203612 10.1021/jacs.9b04974

[17] L. Wang, P. Jiang, Y. Wu, R. Li, M. Chen et al., Co-engineering of *in situ* lithium compensation and oxygen-anchoring modulation enables thermochemically stabilized NCM811-LATP composite cathodes for ultra-long cyclability. Adv. Funct. Mater. **36**(1), e11681 (2026). 10.1002/adfm.202511681

[18] G. Zhang, X. Wen, Y. Gao, R. Zhang, Y. Huang, Inhibiting voltage decay in Li-rich layered oxide cathode: from O_3_-Type to O_2_-Type structural design. Nano-Micro Lett. **16**(1), 260 (2024). 10.1007/s40820-024-01473-7 10.1007/s40820-024-01473-7 PMC11291833 39085663

[19] Y. Lou, H. Yang, Y. Yu, Interface engineering for heightening anionic redox reversibility of Li-rich layered oxides cathodes: recent advances and perspectives. Adv. Energy Mater. **16**(12), e06755 (2026). 10.1002/aenm.202506755

[20] Y. Lou, Z. Lin, J. Shen, J. Sun, N. Wang et al., Simultaneous regulating the surface, interface, and bulk *via* phosphating modification for high-performance Li-rich layered oxides cathodes. Adv. Mater. **37**(6), 2416136 (2025). 10.1002/adma.202416136 10.1002/adma.202416136 39654372

[21] W. Li, B. Song, A. Manthiram, High-voltage positive electrode materials for lithium-ion batteries. Chem. Soc. Rev. **46**(10), 3006–3059 (2017). 10.1039/c6cs00875e 28440379 10.1039/c6cs00875e

[22] K. Xu, Electrolytes and interphases in Li-ion batteries and beyond. Chem. Rev. **114**(23), 11503–11618 (2014). 10.1021/cr500003w 25351820 10.1021/cr500003w

[23] X. Fan, C. Wang, High-voltage liquid electrolytes for Li batteries: progress and perspectives. Chem. Soc. Rev. **50**(18), 10486–10566 (2021). 10.1039/d1cs00450f 34341815 10.1039/d1cs00450f

[24] Q. Sun, Z. Gong, T. Zhang, J. Li, X. Zhu et al., Molecule-level multiscale design of nonflammable gel polymer electrolyte to build stable SEI/CEI for lithium metal battery. Nano-Micro Lett. **17**(1), 18 (2024). 10.1007/s40820-024-01508-z 10.1007/s40820-024-01508-z PMC11427645 39327336

[25] X. Cao, X. Ren, L. Zou, M.H. Engelhard, W. Huang et al., Monolithic solid–electrolyte interphases formed in fluorinated orthoformate-based electrolytes minimize Li depletion and pulverization. Nat. Energy **4**(9), 796–805 (2019). 10.1038/s41560-019-0464-5

[26] Q. Qiu, Y. Chen, J. Xue, J. Zhu, Y. Fu et al., One-step solvothermal synthesis of spherical spinel type NiFe_2–__*x*_Mn_*x*_O_4_-RGO as high-performance supercapacitor electrodes. Ceram. Int. **43**(2), 2226–2232 (2017). 10.1016/j.ceramint.2016.11.006

[27] J. Shen, Q. Yu, J. Sun, J. Qian, Y. Lou et al., Regulating the Li/Ni mixing ratio to enhance transition metal-lattice oxygen interaction for achieving long life lithium-rich layered oxides. Angew. Chem. Int. Ed. **65**(2), e19458 (2026). 10.1002/anie.202519458 10.1002/anie.202519458 41211846

[28] J. Sun, H. Yang, J. Shen, H. Qi, M. Sun et al., Incorporating a lithium-deficient layer and interfacial-confined catalysis enables the reversible redox of surface oxygen species in lithium-rich manganese-based oxides. Energy Environ. Sci. **18**(9), 4335–4347 (2025). 10.1039/D5EE00430F

[29] V.P. Santos, M.F.R. Pereira, J.J.M. Órfão, J.L. Figueiredo, Catalytic oxidation of ethyl acetate over a cesium modified cryptomelane catalyst. Appl. Catal. B Environ. **88**(3–4), 550–556 (2009). 10.1016/j.apcatb.2008.10.006

[30] D. Luo, X. Ding, X. Hao, H. Xie, J. Cui et al., Ni/Mn and Al dual concentration-gradients to mitigate voltage decay and capacity fading of Li-rich layered cathodes. ACS Energy Lett. **6**(8), 2755–2764 (2021). 10.1021/acsenergylett.1c01215

[31] B. Sayahpour, H. Hirsh, S. Bai, N.B. Schorr, T.N. Lambert et al., Revisiting discharge mechanism of CF_x_ as a high energy density cathode material for lithium primary battery. Adv. Energy Mater. **12**(5), 2103196 (2022). 10.1002/aenm.202103196

[32] Y. Jin, S. Li, A. Kushima, X. Zheng, Y. Sun et al., Self-healing SEI enables full-cell cycling of a silicon-majority anode with a coulombic efficiency exceeding 99.9%. Energy Environ. Sci. **10**(2), 580–592 (2017). 10.1039/c6ee02685k

[33] L. Bai, Y. Xu, Y. Liu, D. Zhang, S. Zhang et al., Metal-organic framework glass stabilizes high-voltage cathodes for efficient lithium-metal batteries. Nat. Commun. **16**, 3484 (2025). 10.1038/s41467-025-58639-z 40216782 10.1038/s41467-025-58639-z PMC11992013

[34] G. Assat, A. Iadecola, D. Foix, R. Dedryvère, J.-M. Tarascon, Direct quantification of anionic redox over long cycling of Li-rich NMC *via* hard X-ray photoemission spectroscopy. ACS Energy Lett. **3**(11), 2721–2728 (2018). 10.1021/acsenergylett.8b01798

[35] M. Sathiya, G. Rousse, K. Ramesha, C.P. Laisa, H. Vezin et al., Reversible anionic redox chemistry in high-capacity layered-oxide electrodes. Nat. Mater. **12**(9), 827–835 (2013). 10.1038/nmat3699 23852398 10.1038/nmat3699

[36] J. Li, W. Li, C. Zhang, C. Han, X. Chen et al., Tuning Li_2_MnO_3_-like domain size and surface structure enables highly stabilized Li-rich layered oxide cathodes. ACS Nano **17**(17), 16827–16839 (2023). 10.1021/acsnano.3c03666 37582222 10.1021/acsnano.3c03666

[37] L. Wang, G. Liu, R. Wang, X. Wang, L. Wang et al., Regulating surface oxygen activity by perovskite-coating-stabilized ultrahigh-nickel layered oxide cathodes. Adv. Mater. **35**(11), 2209483 (2023). 10.1002/adma.202209483 10.1002/adma.202209483 36579784

[38] J. Shen, Y. Lou, J. Sun, H. Li, L. Li et al., Achieving high reversible anionic redox activity of Li-rich layered oxides *via* Mg and Mo co-doping. Adv. Funct. Mater. **35**(36), 2425638 (2025). 10.1002/adfm.202425638

[39] H.Y. Asl, A. Manthiram, Reining in dissolved transition-metal ions. Science **369**(6500), 140–141 (2020). 10.1126/science.abc5454 32646985 10.1126/science.abc5454

[40] Y. Wang, Y. Li, Z. Li, N. Qin, F. Wu et al., Monitoring the local coordination evolutions in Li-rich cathode materials via in situ Raman spectroscopy. ACS Energy Lett. **8**, 4888–4894 (2023). 10.1039/d4ee02511c

[41] J. Zhang, F. Cheng, S. Chou, J. Wang, L. Gu et al., Tuning oxygen redox chemistry in Li-rich Mn-based layered oxide cathodes by modulating cation arrangement. Adv. Mater. **31**(42), 1901808 (2019). 10.1002/adma.201901808 10.1002/adma.201901808 31475397

[42] K. Wang, Y. Chu, Z. Huang, H. Yang, M. Yang et al., Unleashing the kinetic limitation of co-free Li-rich Mn-based cathodes *via* ionic/electronic dual-regulation. Adv. Mater. **37**(33), 2504642 (2025). 10.1002/adma.202504642 10.1002/adma.202504642 40444384

[43] L. Zeng, H. Liang, Y. Wang, X. Ying, B. Qiu et al., Quenching-induced lattice modifications endowing Li-rich layered cathodes with ultralow voltage decay and long life. Energy Environ. Sci. **18**(1), 284–299 (2025). 10.1039/d4ee02511c

