# Supplementary material for: Inhibition of Surface-Originated Degradations in Lithium-Rich Layered Cathode via a Pre-Constructed Carbon/Fluorine-Rich Artificial CEI Layer
Source: Nanomicro Lett. 2026 Jul 10;18:433. doi: 10.1007/s40820-026-02276-8 (PMC13354738; doi:10.1007/s40820-026-02276-8)
Supplement: Supplementary file 1 — Supplementary file1 (DOCX 7637 KB) [file 40820_2026_2276_MOESM1_ESM.docx]

Supporting Information for

**Inhibition of Surface-Originated Degradations in Lithium-Rich Layered Cathode via a Pre-Constructed** **Carbon/Fluorine-Rich Artificial CEI Layer**

He Zhao^1, 2^, Jiaqi Sun^1^, Razium Ali Soomro^2^, Ning Sun^1, 3^, Song Hong^4^ and Bin Xu^1, 2^*

^1^ State Key Laboratory of Organic-Inorganic Composites, Beijing Key Laboratory of Electrochemical Process and Technology for Materials, Beijing University of Chemical Technology, Beijing 100029, P. R. China

^2^ Institute of Advanced Energy Storage Materials and Technologies, College of Chemistry & Chemical Engineering, Yan'an University, Yan'an 716000, P. R. China

^3^ School of Chemical Engineering, Guizhou Minzu University, Guiyang 550025, P. R. China

^4^ Analysis Technology R&D Center, Beijing University of Chemical Technology, Beijing 100029, P. R. China

*Corresponding authors. E-mail: xubin@mail.buct.edu.cn or binxumail@163.com (Bin Xu)

**S1 S1 Experimental Section**

**S1.1 Physical Characterizations**

X-ray diffraction (XRD) patterns were acquired using a Bruker D8-Advance X-ray diffractometer (Bruker, 40 kV, 40 mA) with Cu-Kα radiation (λ = 1.5406 Å). Data were recorded in the 2θ range of 10-80 ° at a scan rate of 2 ° min^-1^. Fourier-transform infrared (FTIR, Thermos IS5) spectroscopy was conducted to probe the surface functional groups of the cathodes. Elemental composition was determined by inductively coupled plasma optical emission spectrometer (ICP-OES, Thermos IRIS Intrepid II). Morphology and elemental distribution of the cathodes were examined using Scanning electron microscopy (SEM, Hitachi, SU8600) and high-resolution transmission electron microscopy (HRTEM, FEI TENCAI G2 20) equipped with an energy dispersive spectrometer (EDS) detector. Atomic-resolution high-angle annular dark field (HAADF) images and electron energy loss spectroscopy (EELS) were acquired on an aberration-corrected JEOL JEM-ARM200F scanning transmission electron microscopy (STEM) operated at 200 kV, equipped with a Schottky cold field emission gun (Cold FEG) and Cs corrector (ASCOR). For the data processing of EELS line spectra, due to the weak elemental signals (especially for F) in the post-cycled cathode, partial overlap among the selected regions of interest (ROIs) was adopted to improve the signal-to-noise ratio and enhance data quality. Surface chemical states and elemental composition were analyzed by X-ray photoelectron spectroscopy (XPS, Thermo ESCALAB 250XI) using monochromatic Al Kα radiation (hυ = 1486.6 eV) and Mg/Al double anode light sources.

Time-of-flight secondary ion mass spectrometry (TOF-SIMS) measurements were performed using a TOF.SIMS 5 instrument (ION-TOF GmbH., Münster, Germany) which was equipped with a bismuth primary ion source and a Cs^+^ sputter source for the detection of negative fragment ions. Analysis areas of 100 × 100 µm were probed with a 30 keV Bi^+^ primary ion beam to acquire 3D reconstructions and 2D top-view maps. The sputtering current was maintained at approximately 69.0 nA, corresponding to a sputtering rate of about 0.17 nm s^-1^ relative to SiO_2_. TOF-SIMS data acquisition and processing were performed by SurfaceLab (version 7.2, ION-TOF GmbH, Münster, Germany). Raman spectroscopy was carried out on a Renishaw inVia Reflex Raman spectrometer with a 532 nm laser excitation source. Hard X-ray absorption spectroscopy (hXAS) measurements were conducted in transmission mode at the Shanghai Synchrotron Radiation Facility (SSRF) in Shanghai, China. Data processing and fitting of the X‑ray absorption near-edge structure (XANES) and extended X-ray absorption fine structure (EXAFS) were performed using the Athena software package^2^. Differential electrochemical mass spectrometry (DEMS) was employed to monitor the gas evolution behavior during the initial charging process, using a Shanghai Linglu QAS 100 DEMS spectrometer. Specially prepared electrodes with a high mass loading (approximately 8 mg cm^-2^) and a diameter of 16 mm were used for the DEMS tests.

For post-mortem analyses, the cycled coin cells were disassembled inside an argon-filled glove box, where the concentrations of the H_2_O and O_2_ were maintained below 0.01 ppm. The retrieved electrodes were gently rinsed with dimethyl carbonate (DMC) to eliminate residual LiPF_6_ lithium salts, followed by drying in a vacuum chamber for subsequent characterization.

**S1.2 Cell Configurations and Electrochemical Measurements**

Cathode slurry was obtained by ball milling the active materials, Super P conductive carbon, and polyvinylidene fluoride (PVDF) binder at a weight ratio of 8:1:1, using N-methyl pyrrolidone (NMP) as the solvent. The homogeneous slurry was then coated on a 16 µm thick Al-foil (Shenzhen Kejing Star Technology Co., Shenzhen) and vacuum-dried at 120 °C overnight. The loading of the active material was controlled at about 1.5 mg cm^-2^. CR2025-type coin half-cells were assembled in an argon-filled glove box with H_2_O and O_2_ content below 0.01 ppm. A lithium metal foil (14 mm in diameter, 1.2 mm thick) was used as the counter electrode, and a Celgard 2500 polypropylene membrane was used as the separator. The electrolyte was 1 M LiPF_6_ dissolved in a mixture of ethylene carbonate (EC), ethyl methyl carbonate (EMC), and DMC with a weight ratio of 1:1:1, except the cell for EELS test, which avoids extraneous fluorine contamination by using a LiClO_4_-based electrolyte (1.0 M LiClO_4_ in EC:EMC:DMC = 1:1:1 vol%) and a lithiated polyacrylic acid (PAALi) binder. The electrolyte was purchased from Suzhou Duoduo Chemical Technology Co., Ltd, and used as received.

Full cells were assembled with the as-prepared LNM or CF_x_-LNM as the cathode and graphite as the anode. The graphite anode was prepared by coating a slurry of graphite, Super P conductive carbon, and carboxymethyl cellulose (CMC) binder in a mass ratio of 8:1:1 onto a copper current collector; the active material mass loading was controlled at ~1.0 mg cm^-2^. A commercial 1 M LiPF_6_ dissolved in a mixture of ethylene carbonate (EC), ethyl methyl carbonate (EMC), and DMC with a weight ratio of 1:1:1 was used as the electrolyte, and the electrolyte amount was set at 10 µL per milligram of cathode active material. The N/P ratio was adjusted to ~1.1 based on the specific capacities of the cathode and anode measured at 0.5C. Galvanostatic charge-discharge cycling was carried out in the voltage range of 1.8-4.6 V at room temperature.

Galvanostatic charge-discharge (GCD) tests were carried out using a NEWARE-BTS battery test system. The testing voltage window for half cells was set to 2.0-4.8 V (vs Li/Li^+^). An initial activation cycle was performed at 0.1 C. Galvanostatic intermittent titration technique (GITT) measurements were conducted at 0.1 C with a 0.5 h titration step and a 1 h relaxation step within the 2.0-4.8 V range. For full cell tests, graphite anodes were pre-cycled in half cells at 0.1C for 5 cycles. The negative-to-positive (N/P) ratio was controlled to approximately 1.1, based on the area capacity at 0.5 C. Electrochemical impedance spectroscopy (EIS) tests were performed on a VSP electrochemical workstation (Bio-Logic, France) from 0.1 Hz to 100 kHz and with a 5 mV amplitude. Prior to EIS measurement, the cathodes were fully lithiated by charging to 4.8 V. Cyclic voltammetry (CV) tests were conducted on an electrochemical workstation (AMEITEK, PARSTAT2273) within a potential range of 2.0-4.8 V at a scan rate of 0.2 mV s^-1^. Unless specified otherwise for high-temperature tests, all electrochemical measurements were conducted at 25 °C.

**S2 Supplementary Notes and Discussions**

**S2.1 Formation of the CF_x_-rich artificial CEI layer**

The formation of the CF_x_-rich surface layer is associated with the thermally induced decomposition of TTE on the oxide surface. TTE, with the molecular structure HCF_2_-CF_2_-O-CH_2_-CF_2_-CHF_2_, contains polarized ether C-O bonds and highly fluorinated alkyl segments. Upon thermal treatment, the ether oxygen can coordinate with Lewis-acidic Li^+^/transition-metal sites on the Li-rich cathode surface, which weakens the adjacent C-O bonds and promotes homolytic or heterolytic bond cleavage. This process can generate fluorinated alkoxy and alkyl radicals, such as HCF_2_CF_2_O•, •CH_2_CF_2_CHF_2_ and related fragments. These radical intermediates may further undergo β-scission, dehydrofluorination and fluorine-transfer reactions, producing reactive fluorocarbon species including CF_2_, CF_2_=CHF/CF_2_=CF_2_-type fragments, •CF_2_-containing radicals, and etc. The generated fluorocarbon radicals and unsaturated fluorocarbon fragments can recombine, polymerize or graft onto the cathode surface. As a result, a fluorocarbon-like CEI layer enriched with -CF_x_- units or terminal/branch groups are formed. Therefore, the observed -CF and -CF_2_ signals can be rationalized by the surface-assisted thermal fragmentation of TTE followed by radical polymerization/crosslinking and partial fluorination on the cathode surface. The relevant reaction pathways are shown as follows:

Step 1: ether bond activation

HCF_2_CF_2_-O-CH_2_CF_2_CHF_2_→HCF_2_CF_2_O∙+∙CH_2_CF_2_CHF_2_

or

HCF_2_CF_2_-O-CH_2_CF_2_CHF_2_→HCF_2_CF_2_∙+∙OCH_2_CF_2_CHF_2_

Step 2: cleavage of fluorinated radical and formation of precursors

HCF_2_CF_2_O∙ → ∙CHF_2_ + COF_2_

∙CHF_2_ → :CF_2_ + H∙

HCF_2_CF_2_∙ → CF_2_ = CHF + F∙/H∙

∙CH_2_CF_2_CHF_2_ → CH_2_ = CFCF_2_H + H∙/F∙

Step 3: surface radical crosslinking into CF_x_-Rich artificial CEI

n(:CF_2_) or n(CF_x_ = CF_x_) → −(CF_x_ − CF_x_)_n_ –

**S2.2 Li^+^ transport in the CF_x_-rich artificial CEI**

The artificial CEI formed by the thermal decomposition of TTE is approximately 6 nm thick and amorphous in nature. Such an ultrathin and disordered structure is beneficial for Li-ion transport because it minimizes the diffusion length and avoids the formation of dense crystalline domains or grain-boundary-blocking interfaces that are often present in conventional inorganic coatings. In addition, the fluorinated organic framework contains abundant polar C-F bonds, which may assist local Li-ion hopping through dipole-ion interactions and provide flexible, non-periodic pathways for ion migration. Compared with rigid inorganic artificial CEI layers, such as oxide or phosphate coatings, the CF_x_-rich artificial CEI is expected to be more conformal and mechanically compliant, allowing intimate contact with the cathode surface and better accommodation of surface strain during cycling.

It should also be noted that the high carbon content in this CEI does not necessarily imply high electronic conductivity. The carbon species in the artificial CEI mainly exist as -CF_x_ groups, rather than sp^2^-hybridized carbon networks. Therefore, this layer is fundamentally different from graphitic carbon or hard carbon coatings. Fluorocarbon compounds composed of C-F and C-C σ bonds generally possess low intrinsic electronic conductivity due to the absence of delocalized π electrons. Thus, the CF_x_-rich CEI can electronically passivate the cathode surface and suppress continuous electrolyte oxidation, while its ultrathin amorphous structure still allows Li-ion transport across the interface.

**S3 Supplementary Figures and Tables**


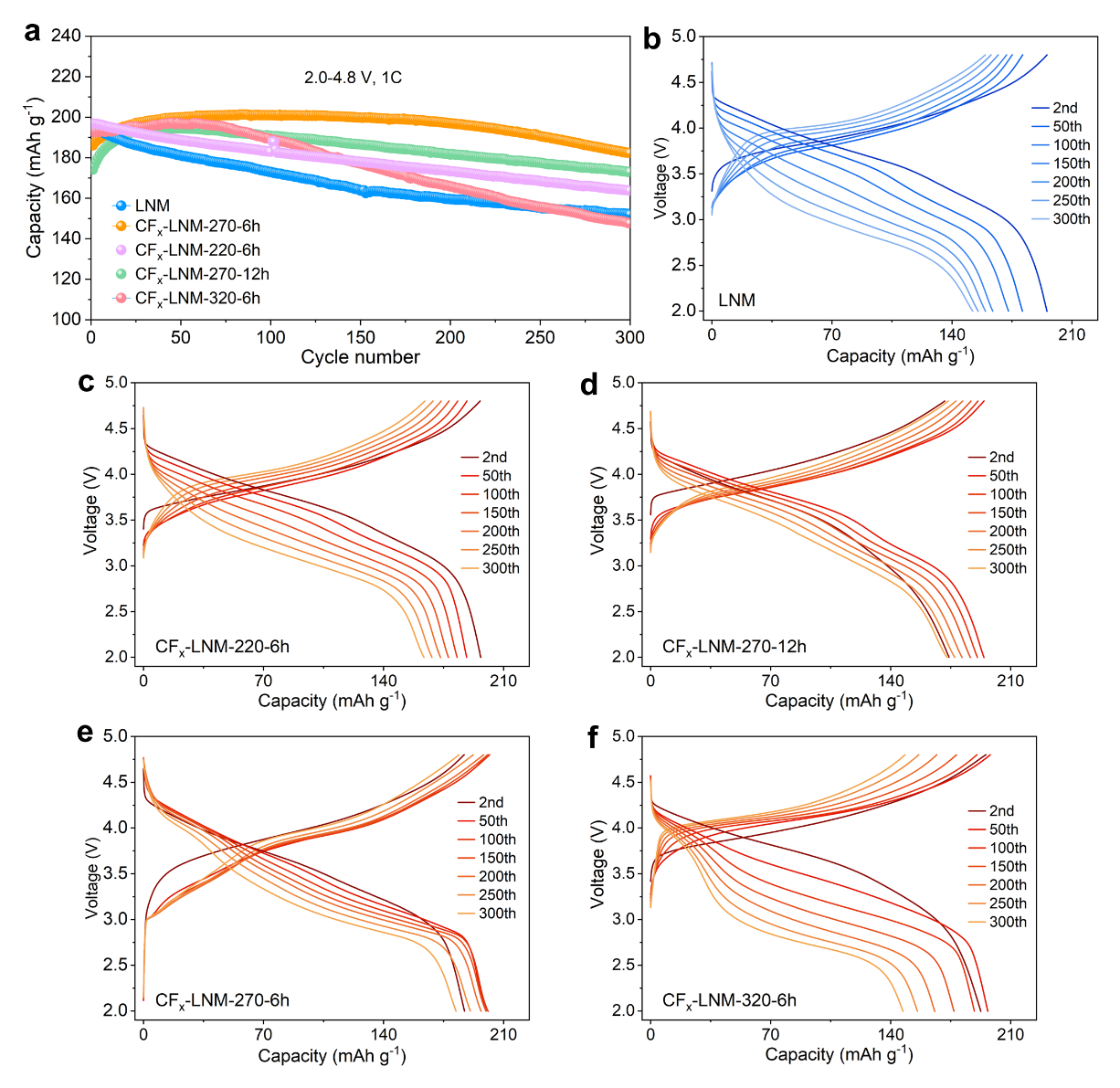


**Fig. S1** Performance comparison of LNM and cathodes with artificial CEI prepared under various conditions: (**a**) overall cycling performances within 300 cycles, and GCD curves for (**b**) LNM, (**c**) CF_x_-LNM-220-6h, (**d**) CF_x_-LNM-270-12h, (**e**) CF_x_-LNM-270-6h, and (**f**) CF_x_-LNM-320-6h.


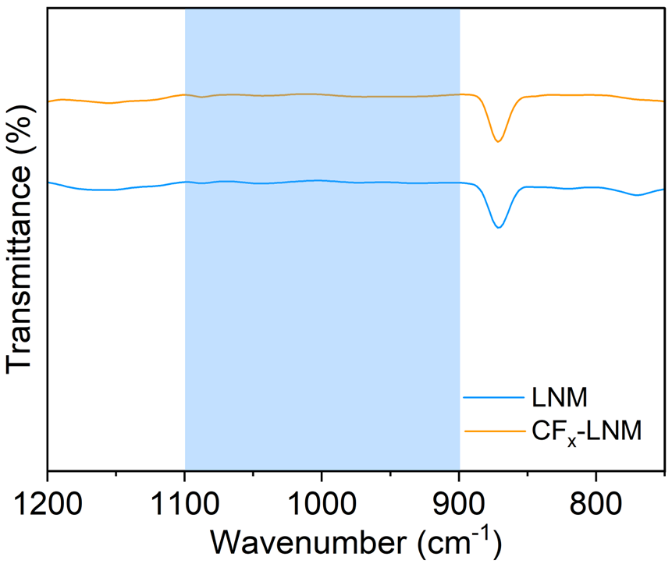


**Fig. S2** Enlarged FT-IR spectra of LNM and CF_x_-LNM.


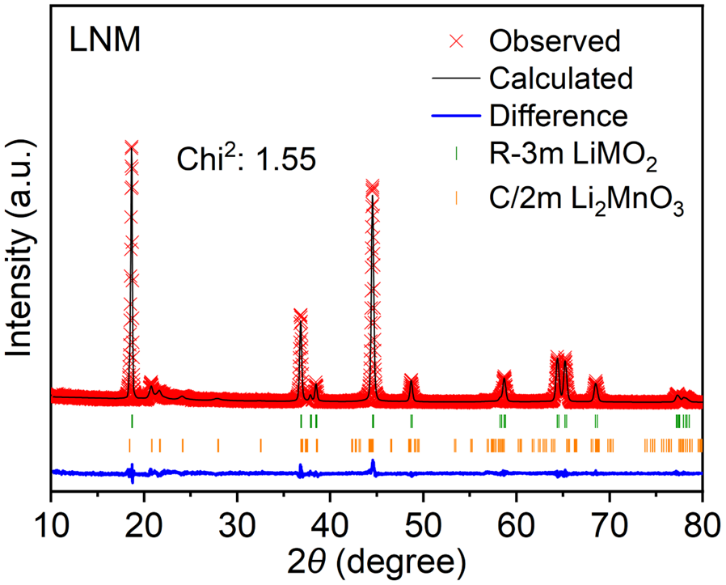


**Fig. S3** XRD pattern and Rietveld refinement result of LNM.


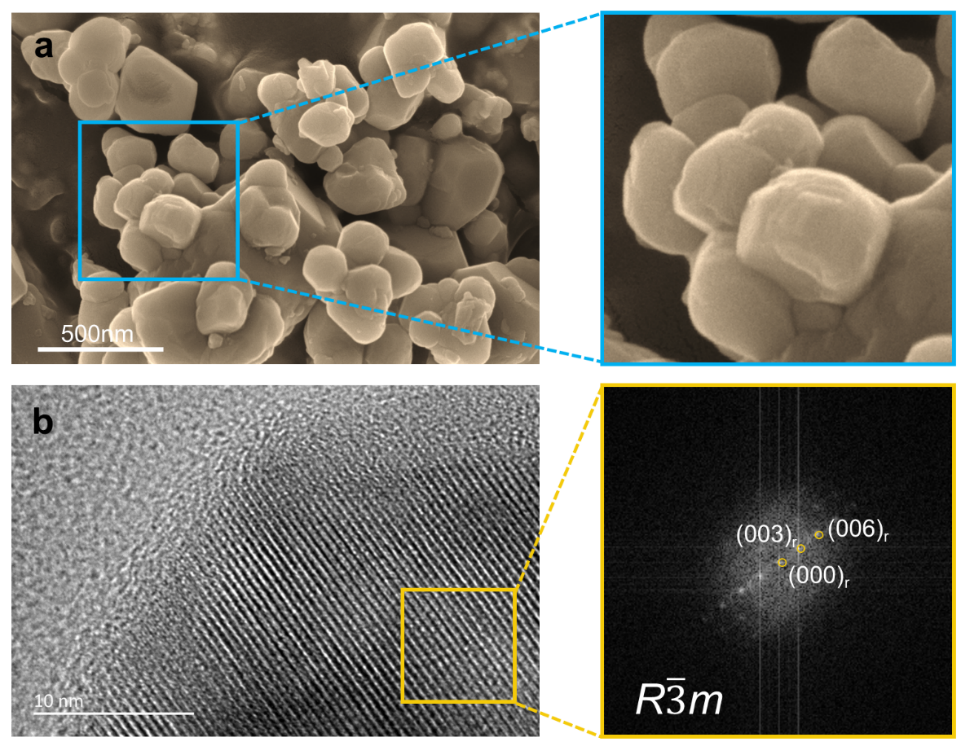


**Fig. S4** (**a**) SEM image and (**b**) HRTEM image and FFT pattern of LNM.


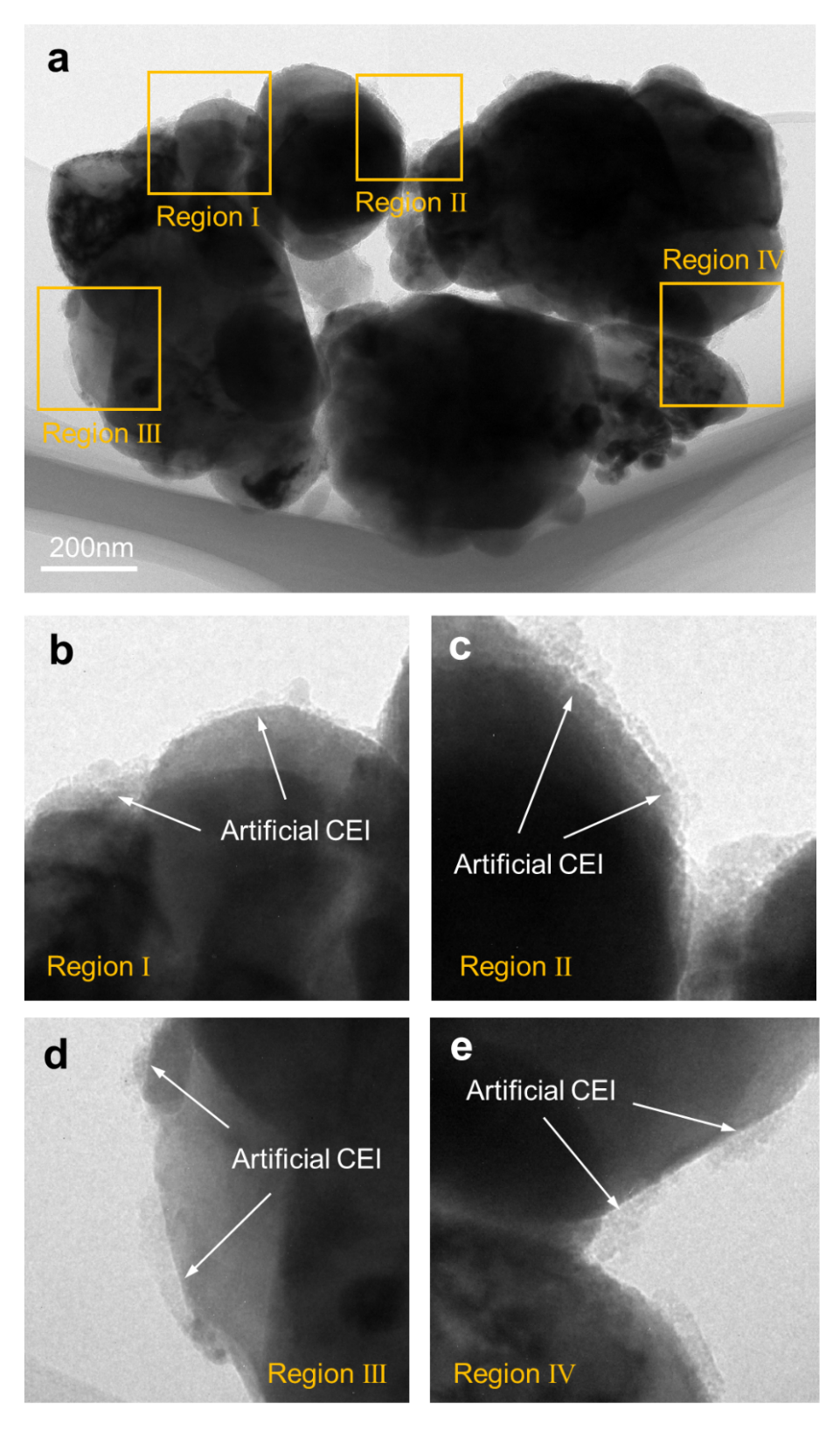


**Fig. S5** (**a**) Low-magnified TEM image of CF_x_-LNM. (**b**-**e**) Enlarged regions in (**a**), showing the uniform distribution of the as-constructed artificial CEI layer.


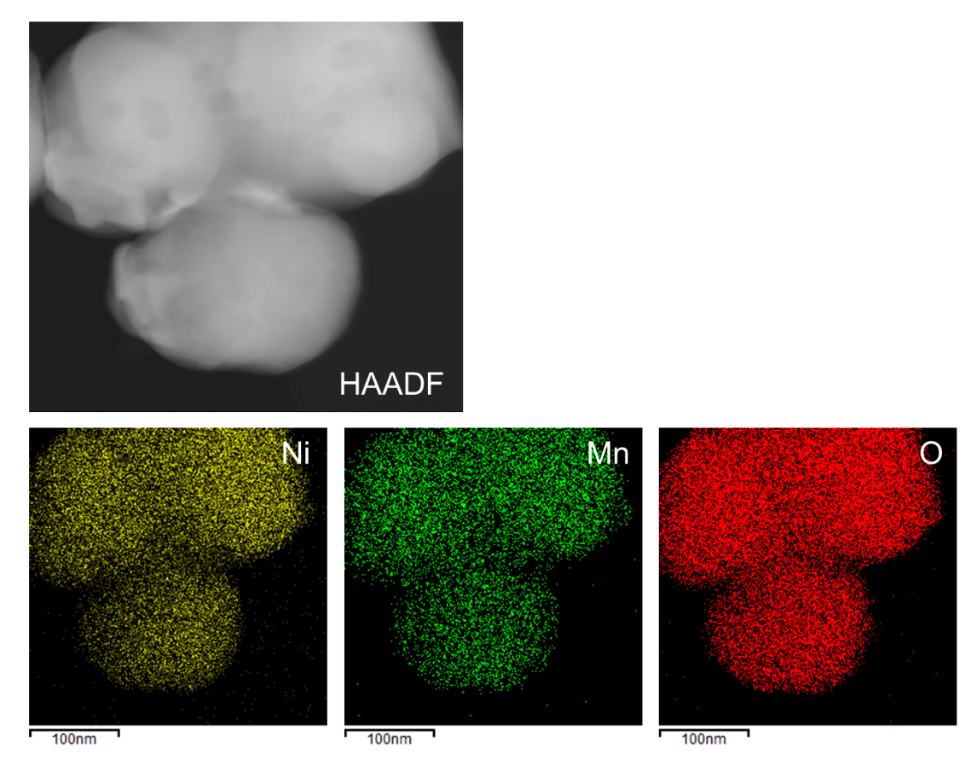


**Fig. S6** HAADF-STEM image and EDS elemental mapping results of LNM, showing the distribution of Ni, Mn, and O.

*
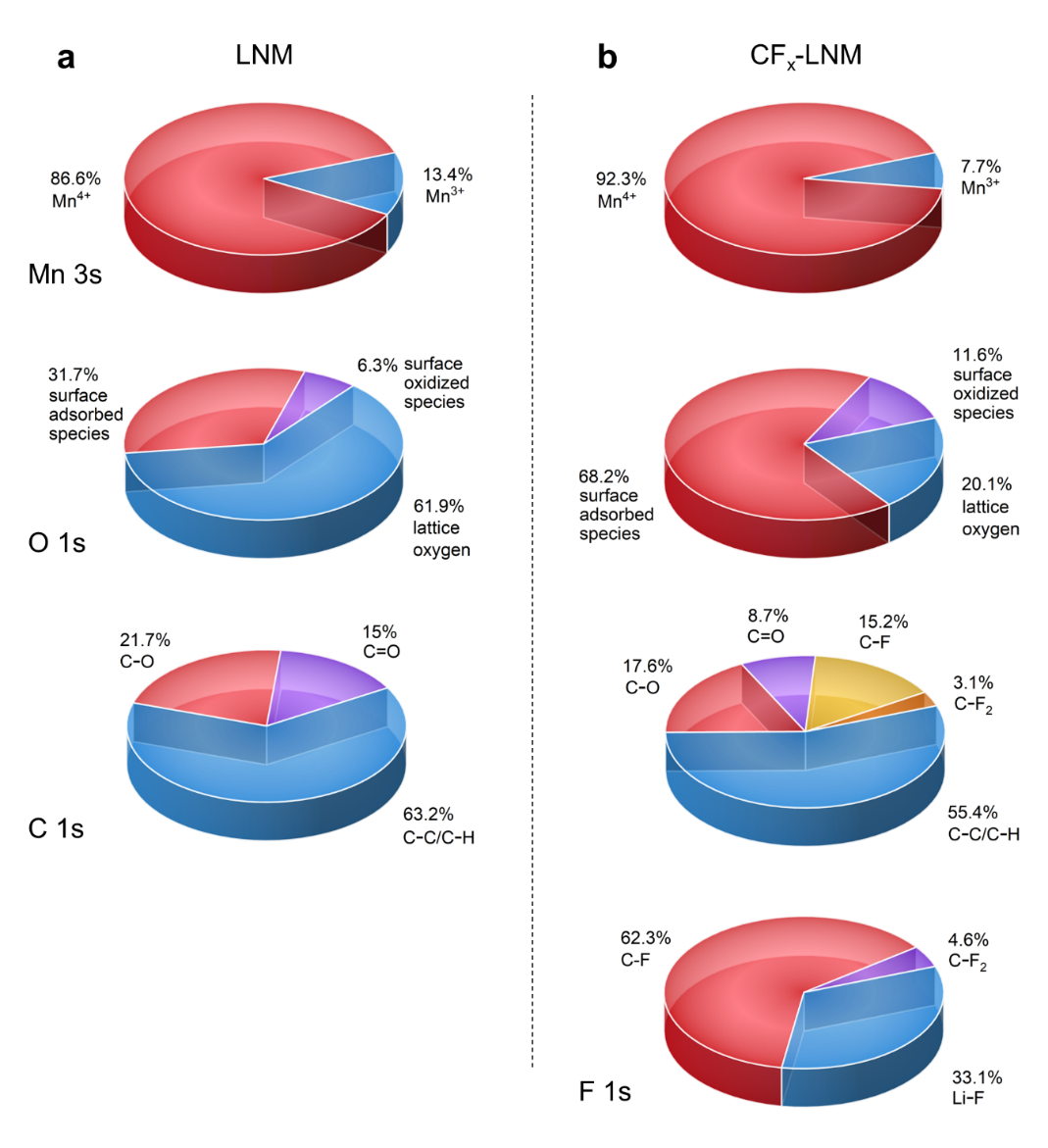
*

**Fig. S7** The proportions of functional groups in the Mn 3s, O1s, C1s, and F1s spectra for (**a**) LNM and (**b**) CF_x_-LNM, calculated based on the XPS deconvolution results.

The Mn valence was calculated based on the splitting energy of 3s orbitals according to the following equation [S1-S2]:

$${Mn}_{(AOS)}=8.956-1.126\Delta E_{s}$$

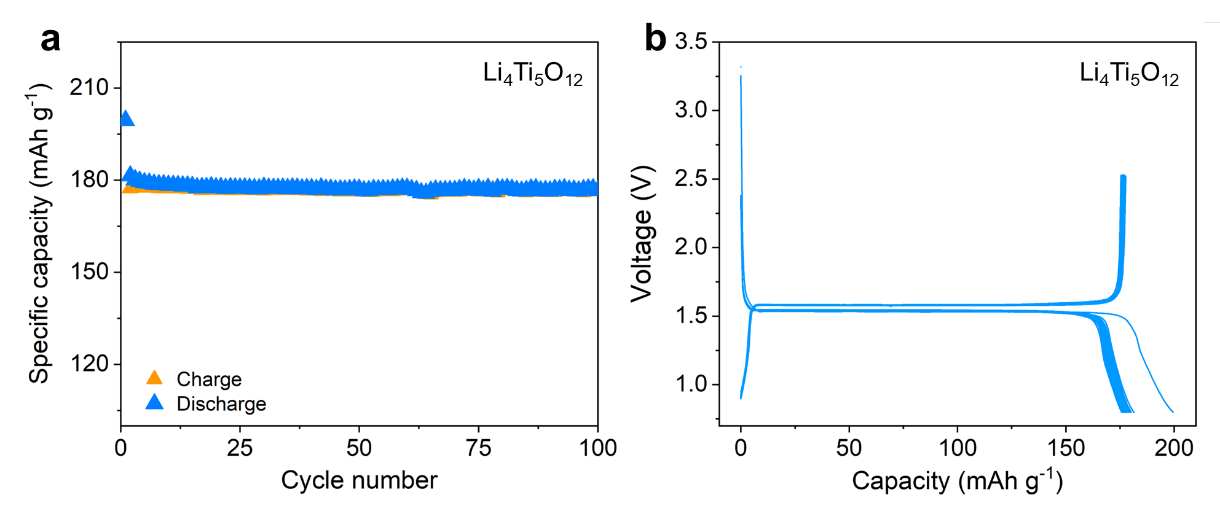


**Fig. S8** Cycling performance of Li_4_Ti_5_O_12_ in half cells at a current rate of 0.5 C within a voltage window of 0.8-2.5 V. The mass loading was controlled at ~2 mg cm^-2^ to match the areal capacity of the cathode. (**a**) Specific capacity as a function of cycle number. (**b**) Galvanostatic charge/discharge curves within 100 cycles.


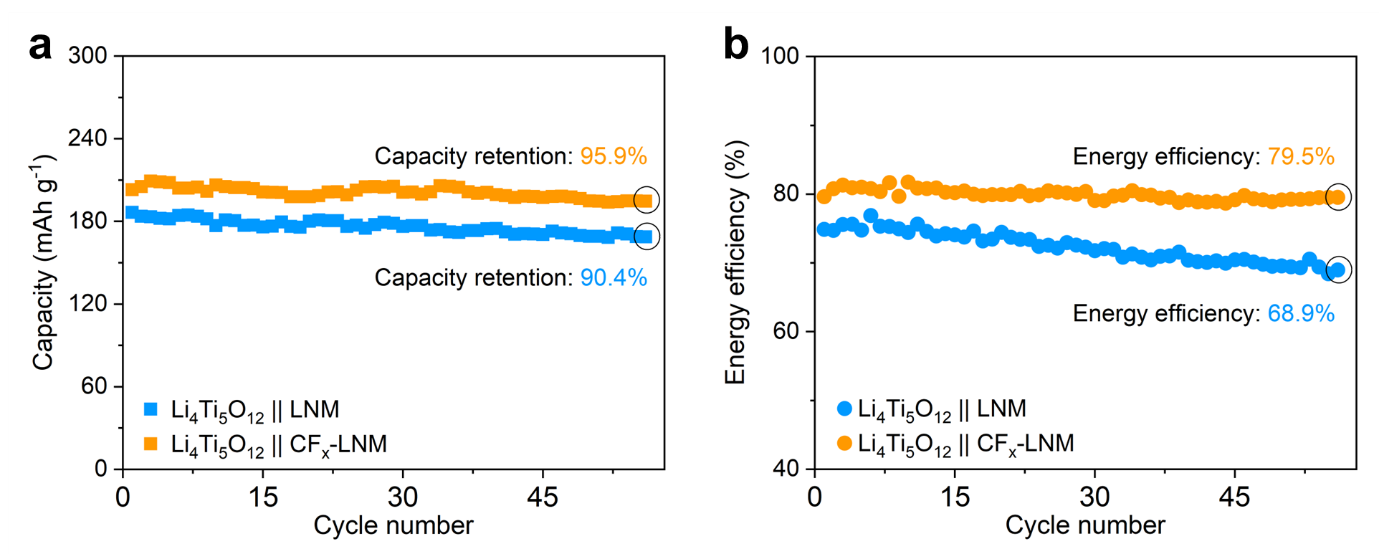


**Fig. S9** Cycling performance of Li_4_Ti_5_O_12_||LNM and Li_4_Ti_5_O_12_||CF_x_-LNM full cell. (**a**) discharge capacity as a function of cycle number. (**b**) Energy efficiency as a function of cycle number.

*
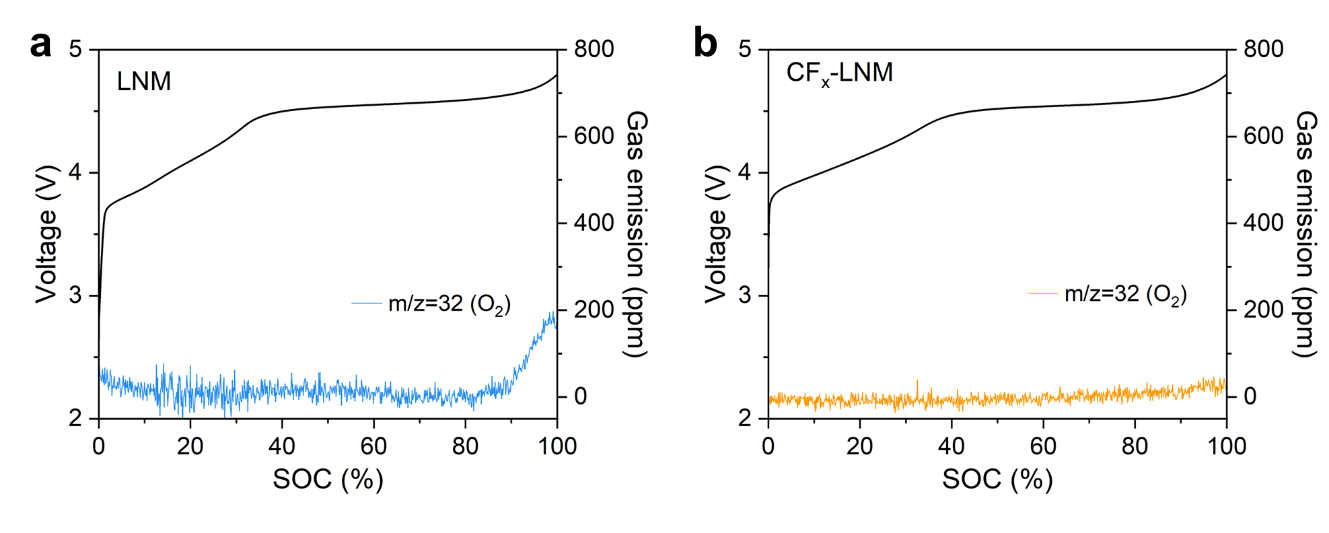
*

**Fig. S10** Differential electrochemical mass spectrometry (DEMS) results for (a) LNM and (**b**) CF_x_-LNM during the initial charging process.


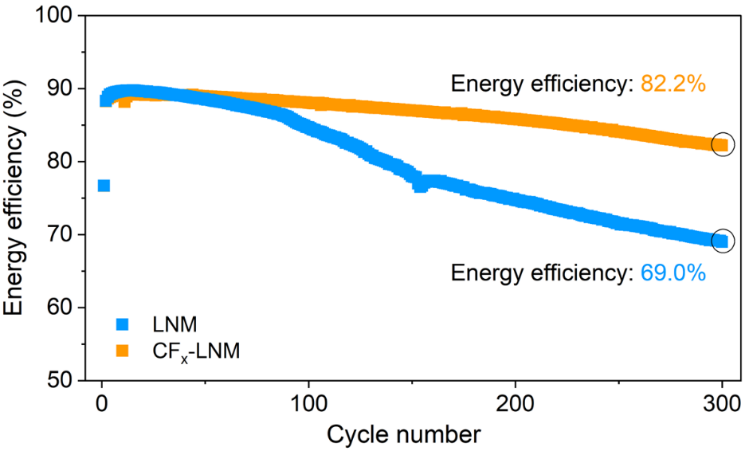


**Fig. S11** Energy efficiency of LNM and CF_x_-LNM half cells within 300 cycles.


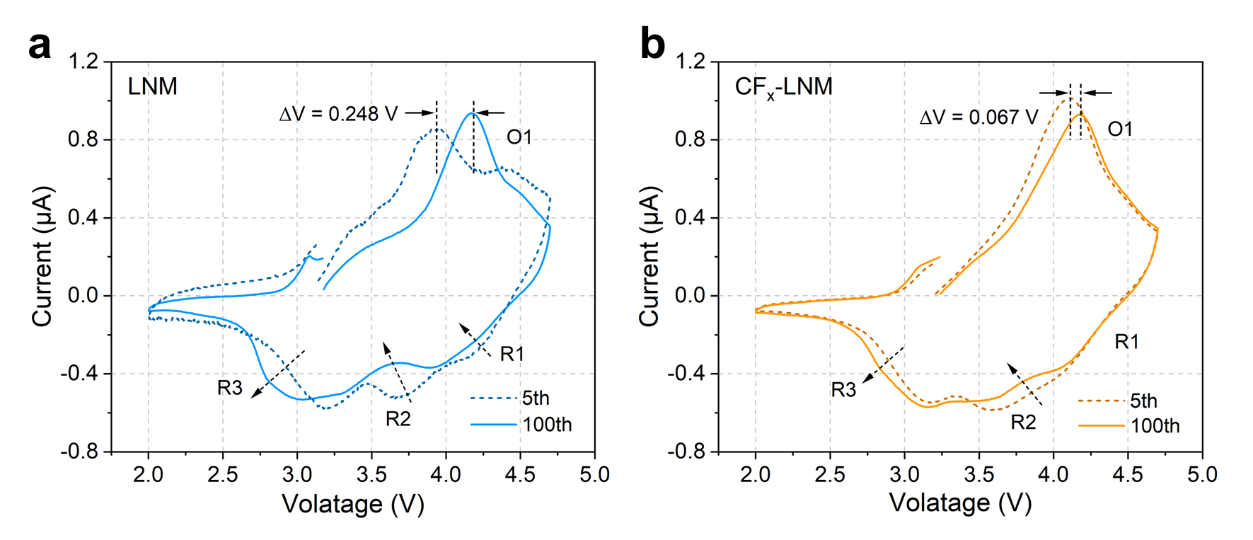


**Fig. S12** Cyclic voltammetry test results of (**a**) LNM and (**b**) CF_x_-LNM. Measurements were performed using a three-electrode cell with a Li metal reference electrode, after 4 and 99 galvanostatic charge-discharge cycles.


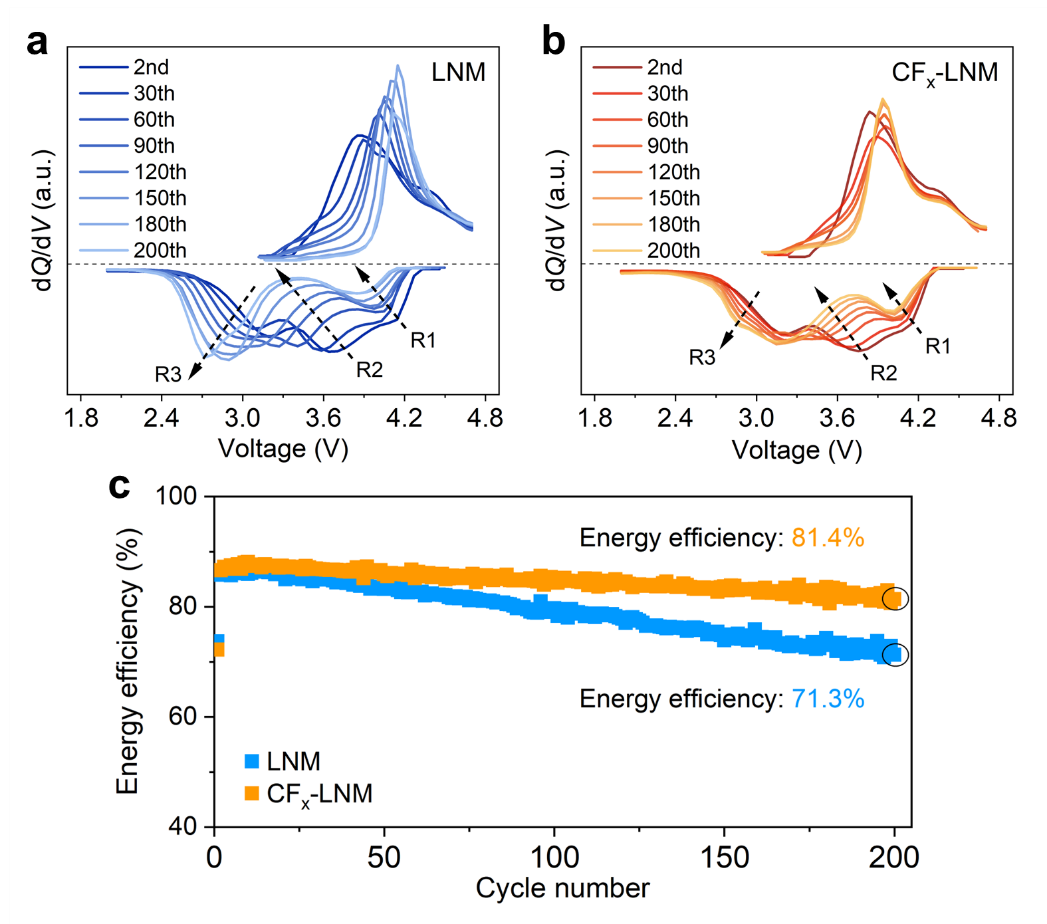


**Fig. S13** dQ/dV curves of (**a**) LNM and (**b**) CF_x_-LNM half cells cycled at 50 ℃. Energy efficiency of LNM and CF_x_-LNM half cells at 50 ℃.


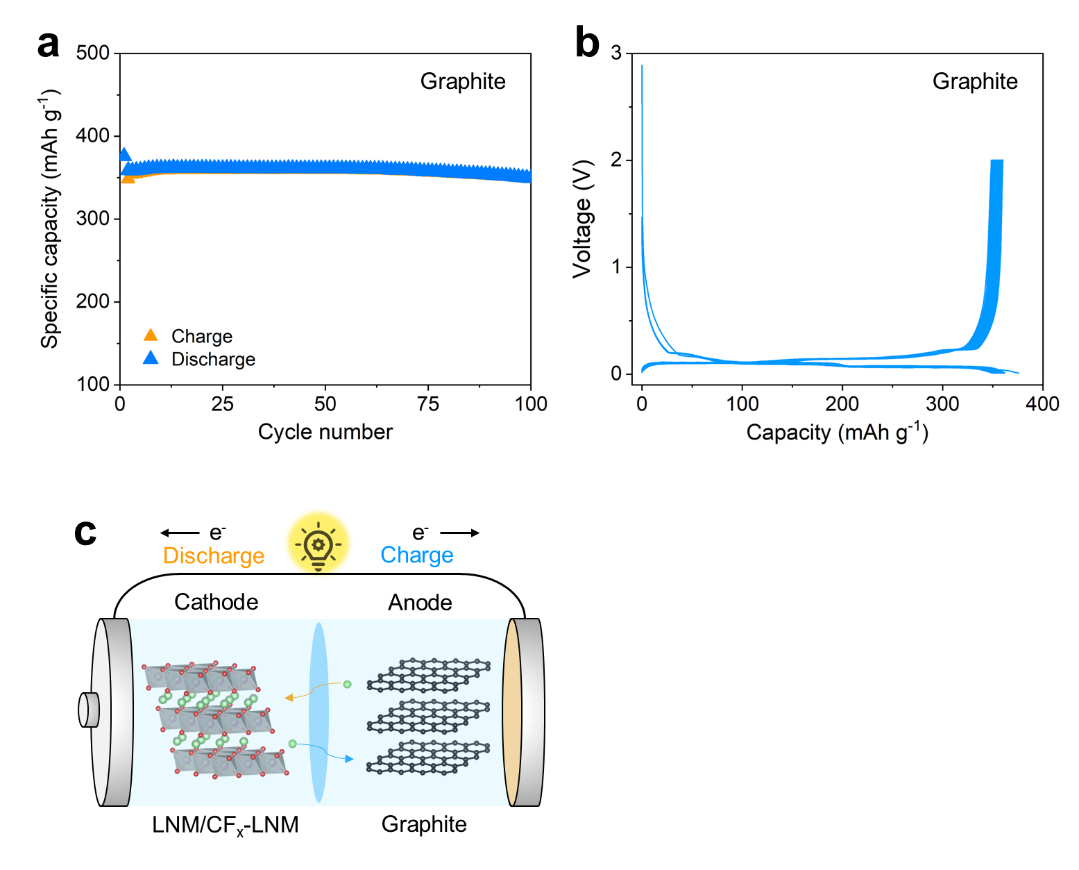


**Fig. S14** Cycling performance of graphite in half cells and the schematic diagram of full cells. (**a**) Specific capacity as a function of cycle number. (**b**) Galvanostatic charge/discharge curves within 100 cycles. (**c**) Schematic diagram of the graphite||LNM and graphite||CF_x_-LNM full cells.


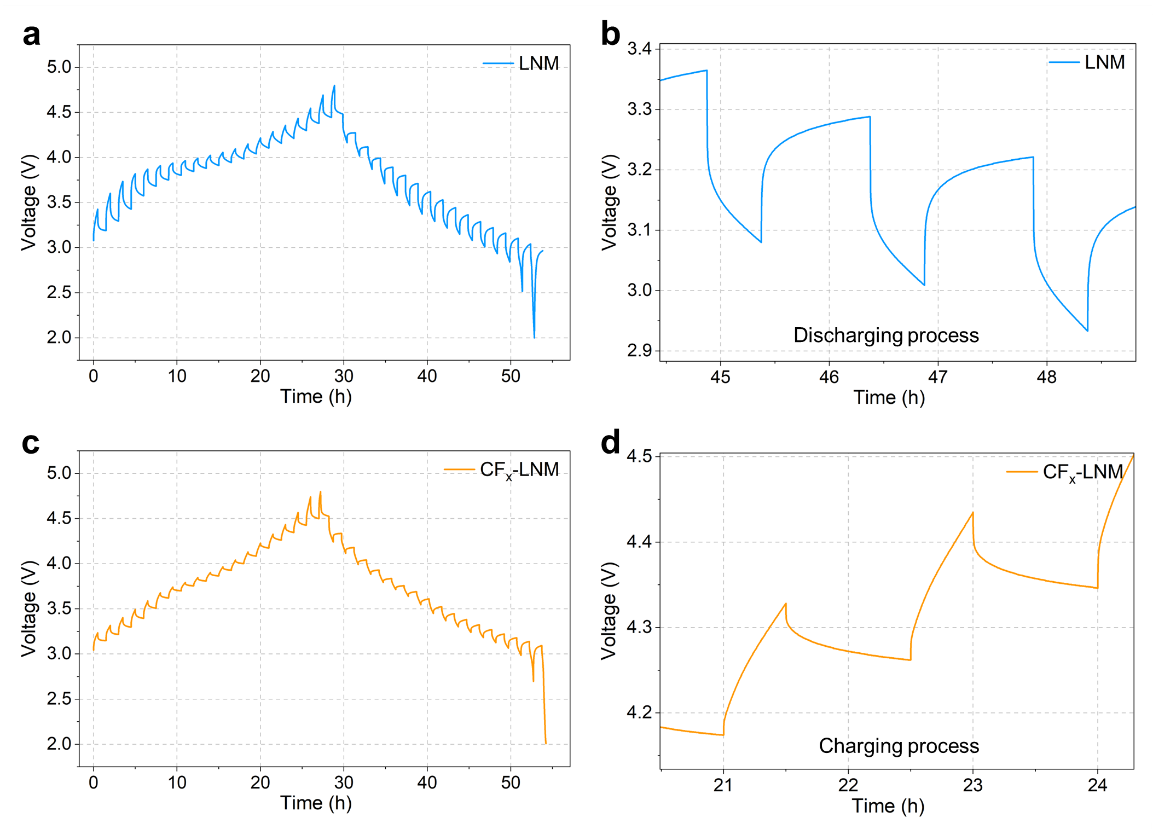


**Fig. S15** GITT plots of (**a** and **b**) LNM and (**c** and **d**) CF_x_-LNM. The cells were cycled at 1 C for 100 cycles before conducted to the GITT test.

In the GITT measurement, the Li^+^ diffusion coefficients ($\text{D}_{\text{Li}^{\text{+}}}$) values were calculated by the following equation [S3-S4]:

| $\text{D}_{\text{Li}^{\text{+}}}\text{=}\frac{\text{4}L^{\text{2}}}{\text{πτ}}\left（ \frac{\text{Δ}\text{E}_{\text{S}}}{\text{Δ}\text{E}_{\text{τ}}} \right）^{\text{2}}\text{ (}\text{τ}\text{≪}\text{ }\text{L}^{\text{2}}\text{/}\text{D}_{\text{Li}^{\text{+}}}\text{)}$ |  |
| --- | --- |

Where *L* is the Li ion diffusion distance at the cathode particle, *τ* stands for the duration of the current pulse, *E_τ_* is the voltage change during the constant current pulse, and *E_s_* represents the steady-state voltage change due to the current pulse.

*
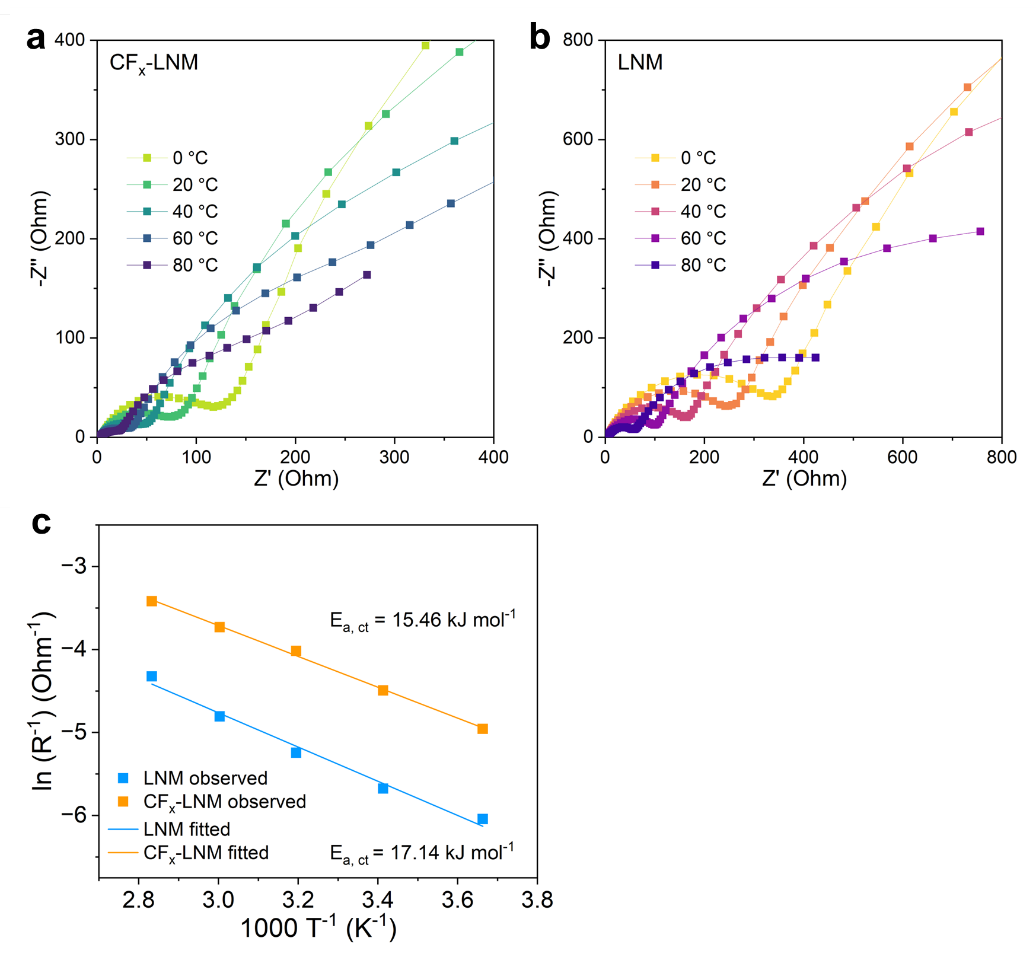
*

**Fig. S16** Temperature-dependent electrochemical impedance spectroscopy (EIS) for (**a**) CF_x_-LNM and (**b**) LNM. (**c**) Comparison of activation energies for charge transfer (CT) in LNM and CF_x_-LNM cells. The data were acquired in half cells at full delithated state (charged to 4.8 V). The activation energies were calculated according to the Temperature-dependent EIS results by the Arrhenius equation [S5-S6]:

$$ln(k)=ln(A)-\frac{E_{a}}{RT}$$

Where*k* stands for the reaction rate constant, *A* is the pre-exponential factor, *Eₐ* is the apparent activation energy for the process, *R* is the ideal gas constant, and *T* is the absolute temperature.


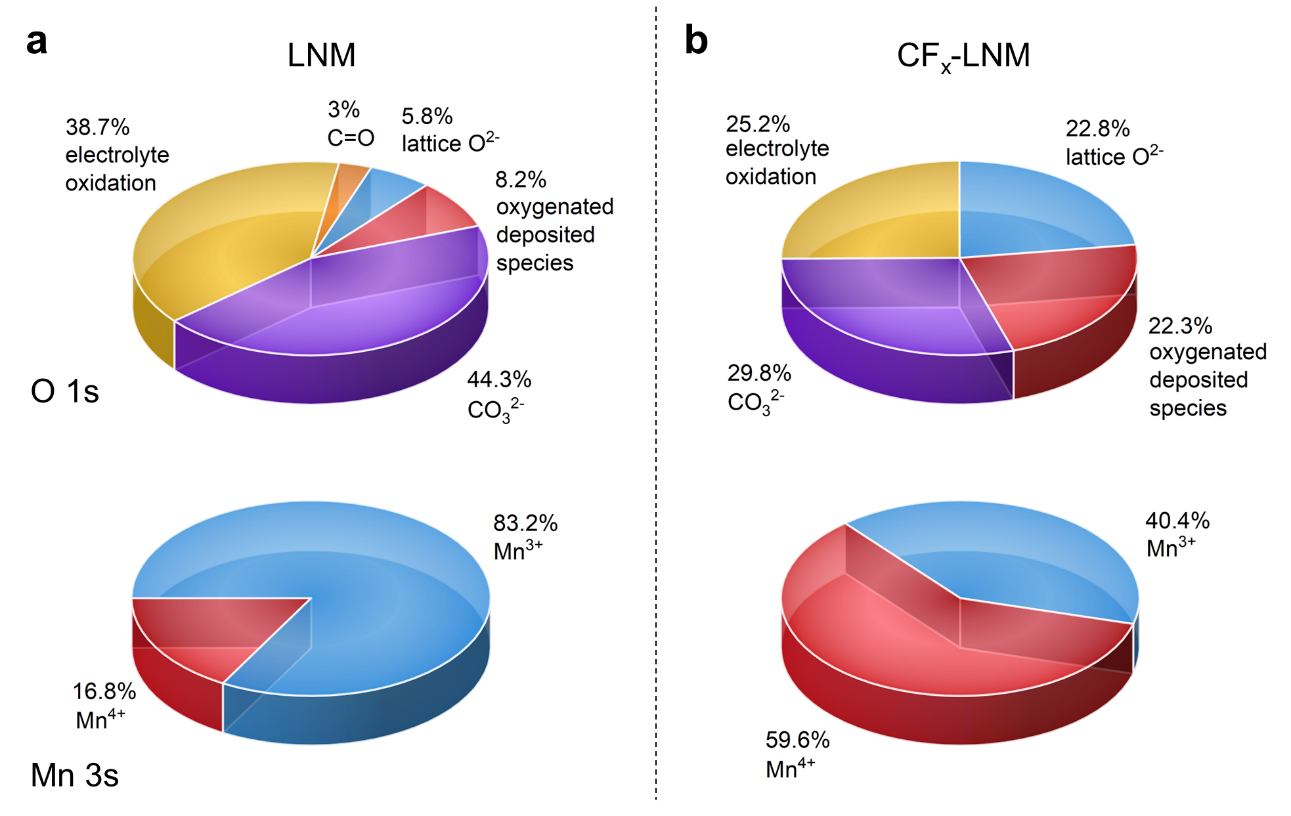


**Fig. S17** The proportions of functional groups in the O1s and Mn 3s spectra for (a) LNM and (b) CF_x_-LNM, calculated based on the XPS deconvolution results.


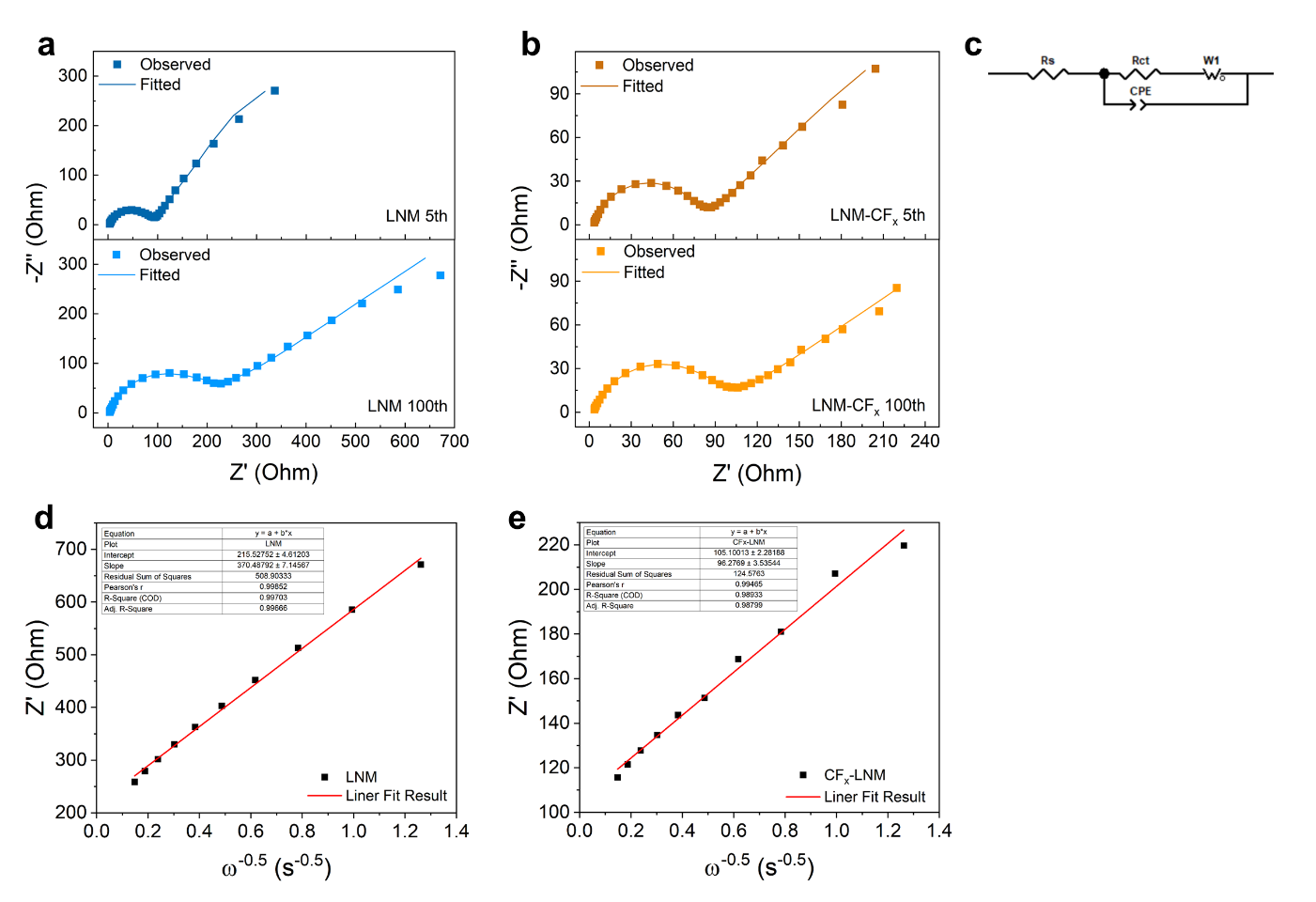


**Fig. S18** EIS results for (**a**) LNM and (**b**) CF_x_-LNM after 5 and 100 cycles. (**c**) Equivalent circuit for the EIS fitting. Fitted Li^+^ diffusion coefficient (*D*_Li+_) results for (**d**) LNM and (**e**) CF_x_-LNM according to the Warburg impedance.


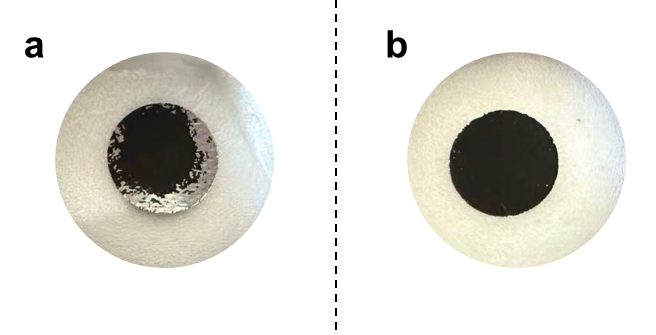


**Fig. S19** Optical images of the electrodes extracted from cycled (**a**) LNM and (**b**) CF_x_-LNM half cells, showing the physical statues of the electrodes.


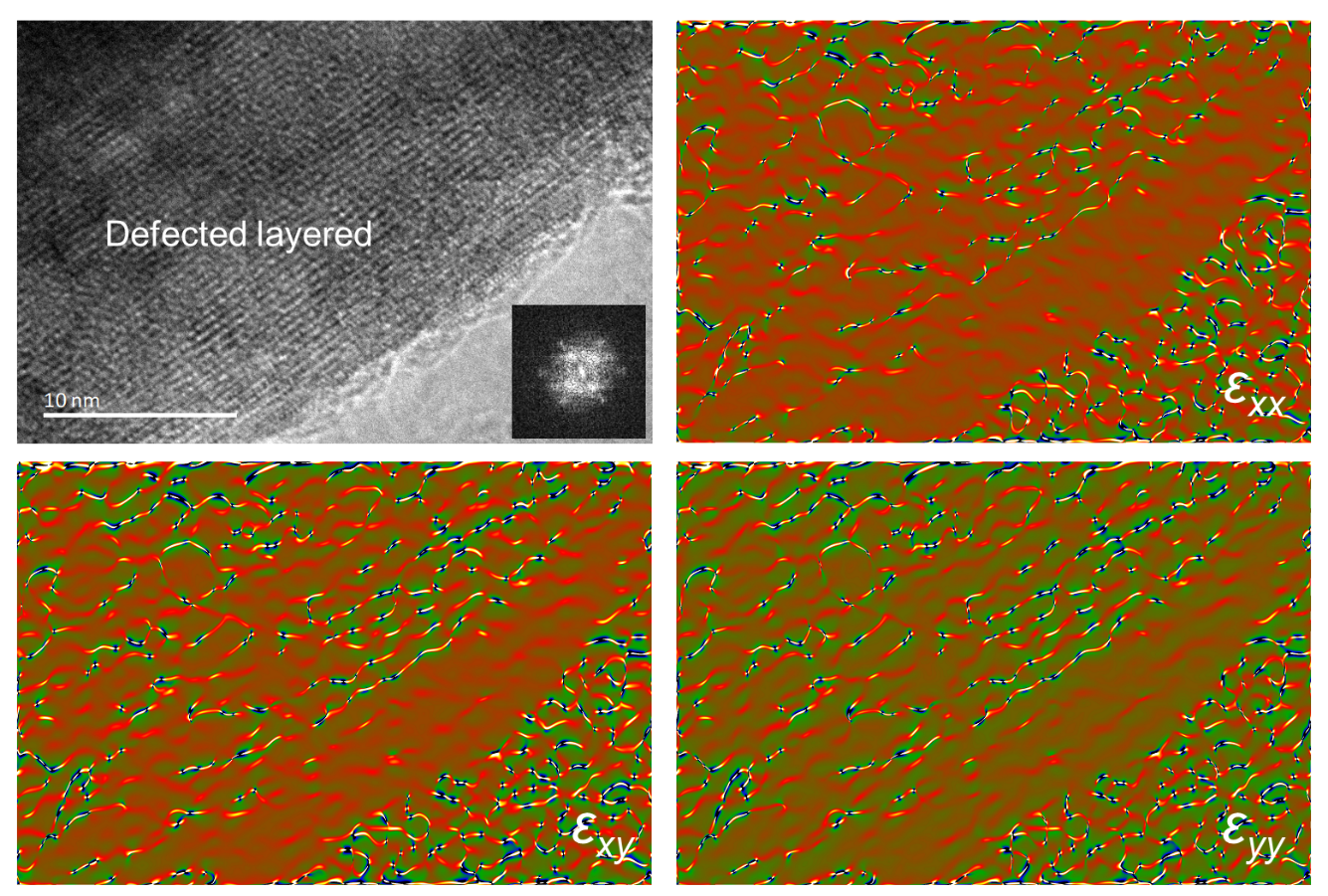


**Fig. S20** HRTEM image and GPA results for post-cycled LNM.

**Table S1** The cycling performance of cathodes at 1 C, room temperature

| Sample | Capacity (mAh g^−1^) | | Capacity retention (%)* | Electrochemical activated |
| --- | --- | --- | --- | --- |
|  | Initial | After 300^th^ |  |  |
| LNM | 196.8 | 152.1 | 77.3 | No |
| CF_x_-LNM-270 | 186.1 | 182.3 | 90.6 | Yes |
| CF_x_-LNM-200 | 196.7 | 163.7 | 83.2 | No |
| CF_x_-LNM-250 | 176.2 | 173.0 | 88.9 | Yes |
| CF_x_-LNM-320 | 193.2 | 147.6 | 74.9 | Yes |

* Calculated based on the highest discharge capacity among 300 cycles.

**Table S2** Rietveld refinement results of LNM, based on two-phase model.

Space group: *R-3m*, LiNi_0.5_Mn_0.5_O_2_, a=b=2.8594 Å, c=14.2493 Å, fraction: 68.28%

| Atom | Wyckoff position | x | y | z | Occupancy |
| --- | --- | --- | --- | --- | --- |
| L1 | 3a | 0 | 0 | 0 | 0.09620 |
| Ni1 | 3a | 0 | 0 | 0 | 0.00380 |
| Ni2 | 3b | 0 | 0 | 0.50000 | 0.01287 |
| Li2 | 3b | 0 | 0 | 0.50000 | 0.00380 |
| Mn | 3b | 0 | 0 | 0.50000 | 0.05000 |
| O | 6c | 0 | 0 | 0.24320 | 0.16667 |

Space group: *C/2m*, Li_2_MnO_3_, a= 4.9453 Å, b= 8.5413 Å, c= 5.0967 Å, fraction: 31.72%

| Atom | Wyckoff position | x | y | z | Occupancy |
| --- | --- | --- | --- | --- | --- |
| Mn1 | 4g | 0 | 0.16708 | 0 | 0.50000 |
| Li1 | 4g | 0 | 0.50000 | 0 | 0.25000 |
| Li2 | 2b | 0 | 0.5 | 0 | 0.25000 |
| Li3 | 2c | 0 | 0.66060 | 0.50000 | 0.50000 |
| O1 | 4i | 0.14432 | 0 | 0.22730 | 0.5000 |
| O2 | 8j | 0.25389 | 0.28236 | 0.22330 | 1.00000 |

**Table S3** Rietveld refinement results of CF_x_-LNM, based on two-phase model.

Space group: *R-3m*, LiNi_0.5_Mn_0.5_O_2_, a=b=2.8594 Å, c=14.2491 Å, fraction: 67.58%

| Atom | Wyckoff position | x | y | z | Occupancy |
| --- | --- | --- | --- | --- | --- |
| L1 | 3a | 0 | 0 | 0 | 0.09735 |
| Ni1 | 3a | 0 | 0 | 0 | 0.00265 |
| Ni2 | 3b | 0 | 0 | 0.50000 | 0.01402 |
| Li2 | 3b | 0 | 0 | 0.50000 | 0.00265 |
| Mn | 3b | 0 | 0 | 0.50000 | 0.05000 |
| O | 6c | 0 | 0 | 0.24293 | 0.16667 |

Space group: *C/2m*, Li_2_MnO_3_, a=4.9504 Å, b=8.5418 Å, c=5.0888 Å, fraction: 32.42%

| Atom | Wyckoff position | x | y | z | Occupancy |
| --- | --- | --- | --- | --- | --- |
| Mn1 | 4g | 0 | 0.16708 | 0 | 0.50000 |
| Li1 | 4g | 0 | 0.50000 | 0 | 0.25000 |
| Li2 | 2b | 0 | 0.5 | 0 | 0.25000 |
| Li3 | 2c | 0 | 0.66060 | 0.50000 | 0.50000 |
| O1 | 4i | 0.14432 | 0 | 0.22730 | 0.5000 |
| O2 | 8j | 0.25389 | 0.28236 | 0.22330 | 1.00000 |

**Table S4** EDS mapping results of LNM and CF_x_-LNM, corresponding to Figure 1f and S5

| Cathode | Element | Atomic proportion (%) |
| --- | --- | --- |
| LNM | O | 73.2 |
|  | Mn | 20.4 |
|  | Ni | 6.5 |
| CF_x_-LNM | O | 71.1 |
|  | Mn | 16.5 |
|  | Ni | 5.4 |
|  | F | 2.1 |
|  | C | 4.9* |

*Owing to the presence of the carbon support film in the TEM specimen holder, this value may be higher than actual.

**Table S5** The cycling performance of LNM and CF_x_-LNM at 1 C, room temperature.

| Sample | Capacity (mAh g^−1^) | | Capacity retention (%) | Voltage fade (mV cycle^−1^) | Energy retention (%) |
| --- | --- | --- | --- | --- | --- |
|  | Initial | After 300^th^ |  |  |  |
| LNM | 196.8 | 152.1 | 77.3* | 1.9 | 65.0 |
| CF_x_-LNM | 186.1 | 182.3 | 90.6* | 0.88 | 85.4 |

* Calculated based on the highest discharge capacity among 300 cycles.

**Table S6** The fitted charge transfer resistance (R_CT_) for LNM and CFx-LNM under different testing temperature

| Temperature (℃) | R_CT_ for LNM (Ohm) | R_CT_ for CF_x_-LNM (Ohm) |
| --- | --- | --- |
| 0 | 421.2 | 142.1 |
| 20 | 291.6 | 89.4 |
| 40 | 189.5 | 55.5 |
| 60 | 122.3 | 41.7 |
| 80 | 75.5 | 30.5 |

**Table S7** The fitted structural parameters derived from the Mn K-edge EXAFS fitting in Fig.6, including the Mn-O bond length, the Debye-Waller factor (σ^2^), and the R-facto

| Cathode | Mn-O distance (Å) | σ^2^ (Å^2^) | R |
| --- | --- | --- | --- |
| Pristine | 1.91374 | 0.00047 | 0.013 |
| LNM | 1.91148 | 0.00146 | 0.021 |
| CF_x_-LNM | 1.91546 | 0.00128 | 0.018 |

**Supplementary References**

1. V.P. Santos, M.F.R. Pereira, J.J.M. Órfão, J.L. Figueiredo, Catalytic oxidation of ethyl acetate over a cesium modified cryptomelane catalyst. Appl. Catal. B Environ. **88**(3–4), 550–556 (2009). <https://doi.org/10.1016/j.apcatb.2008.10.006>
2. H. Zhao, W. Li, J. Li, H. Xu, C. Zhang et al., Enhance performances of co-free Li-rich cathode by eutesctic melting salt treatment. Nano Energy **92**, 106760 (2022). <https://doi.org/10.1016/j.nanoen.2021.106760>
3. Z. Zhu, D. Yu, Y. Yang, C. Su, Y. Huang et al., Gradient Li-rich oxide cathode particles immunized against oxygen release by a molten salt treatment. Nat. Energy **4**(12), 1049–1058 (2019). <https://doi.org/10.1038/s41560-019-0508-x>
4. Y. Lou, Z. Lin, J. Shen, J. Sun, N. Wang et al., Simultaneous regulating the surface, interface, and bulk *via* phosphating modification for high-performance Li-rich layered oxides cathodes. Adv. Mater. **37**(6), 2416136 (2025). <https://doi.org/10.1002/adma.202416136>
5. L. Bai, Y. Xu, Y. Liu, D. Zhang, S. Zhang et al., Metal-organic framework glass stabilizes high-voltage cathodes for efficient lithium-metal batteries. Nat. Commun. **16**, 3484 (2025). <https://doi.org/10.1038/s41467-025-58639-z>
6. L. Zeng, Y. Hu, C. Lu, G. Pan, M. Li, Arrhenius equation-based model to predict lithium-ions batteries’ performance. J. Mar. Sci. Eng. **10**(10), 1553 (2022). <https://doi.org/10.3390/jmse10101553>
